# R pyocin tail fiber structure reveals a receptor-binding domain with a lectin fold

**DOI:** 10.1371/journal.pone.0211432

**Published:** 2019-02-05

**Authors:** Adam J. Salazar, Mukul Sherekar, Jennifer Tsai, James C. Sacchettini

**Affiliations:** Department of Biochemistry and Biophysics, Texas A&M University, College Station, TX, United States of America; University of Liverpool, UNITED KINGDOM

## Abstract

R pyocins are ɸCTX-like myophage tailocins of *Pseudomonas sp*. Adsorption of R pyocins to target strains occurs by the interaction of tail fiber proteins with core lipopolysaccharide (LPS). Here, we demonstrate that N-terminally truncated R pyocin tail fibers corresponding to a region of variation between R-subtypes are sufficient to bind target strains according to R-subtype. We also report the crystal structures of these tail fiber proteins and show that they form an elongated helical trimer composed of three domains arranged linearly from N- to C-terminus: a baseplate proximal head, medial shaft, and distal foot. The head and shaft domains contain novel structural motifs. The foot domain, however, is composed of a conserved jellyroll fold and shares high structural similarity to the tail fiber of myophage AP22, podophage tailspike C-terminal domains (LKA-1 and ɸ297), and several eukaryotic adhesins (discoidin I/II, agglutinin, and octocoral lectin). Many of these proteins bind polysaccharides by means of their distal loop network, a series of highly variable loops at one end of the conserved jellyroll fold backbone. Our structures reveal that the majority of R-subtype specific polymorphisms cluster in patches covering a cleft formed at the oligomeric interface of the head domain and in a large patch covering much of the foot domain, including the distal loop network. Based on the structural variation in distal loops within the foot region, we propose that the foot is the primary sugar-binding domain of R pyocins and R-subtype specific structural differences in the foot domain distal loop network are responsible for binding target strains in an R-subtype dependent manner.

## Introduction

Discovered in 1954 by François Jacob, pyocins are highly diverse peptide inhibitors of pseudomonad growth [1–3]. They include S, F, L, and R-types, which are further classified into subtypes by strain sensitivity [1, 4–7]. Of these, F and R pyocins resemble phage tail assemblies in both structure and function, and are more accurately categorized as “tailocins”. Tailocins are prophages that lack head structural genes and genome packaging mechanisms, but retain conserved elements of phage tails, including the inner tube, outer sheath, baseplate, and tail fibers [1, 4]. F pyocins are closely related to lambda-like siphophages by morphology and genomic organization, and contain flexible, non-contractile tails [1, 5]. R pyocins, on the other hand, are related to the P2-like myophage ɸCTX, and contain rigid, contractile tails [1]. They are also auspiciously resistant to protease treatment, temperatures up to 60°C, and inactivation by sterilizing UV exposure [8].

The primary cell anchoring protein of myophages and myophage tailocins is the tail fiber [1, 4, 9, 10]. Many myophage tail fibers, including those from R pyocins, require one or more chaperones for efficient assembly, which are often co-expressed, adjacent, and downstream of the tail fiber [4, 10, 11]. Tail fibers belonging to T-even myophages are trimeric, linearly assembling complexes of proteins with one or more globular knots separated by regions of fibrous character [11]. In the case of model T-even bacteriophage T4, separate baseplate proximal (gp34), medial (gp35 and gp36), and distal (gp37) tail fiber proteins compose a single long tail fiber (LTF) complex [10]. In T-even myophages, only the distal long tail fiber or a capping adhesin is responsible for receptor binding [11, 12]. Unlike T-even myophages, P2-like myophages and P2-like tailocin tail fibers consist of a single, large, trimeric tail fiber [5, 12]. These fibers contain a conserved N-terminal DUF3751 collar-tail domain absent in T-even myophages [4]. The location of this domain at the fiber N-terminus suggests that it is responsible for mediating contact of the tail fiber to the base plate apparatus and triggering tail tube injection, functions analogous to the T4 collar-tail protein, gp34 [4, 10].

Myophage tail fibers are capable of binding cell surface peptides and LPS components, including O-antigen, outer core, and inner core LPS [10, 11]. R pyocins, however, have only been confirmed to bind outer core LPS and not a peptide target [4, 13, 14]. Specifically, it is known that R1-subtype pyocins require the presence of common Pseudomonad outer core L-rhamnose to adsorb, whereas R2-subtype and R5-subtype pyocins require inferior outer core α or β-glucoses [14, 15]. Although the minimal tail fiber fragment required for specific binding has not been identified, target strain sensitivity switching has been demonstrated by substitution of the region downstream of the DUF3751 domain extending to the C-terminus with other R pyocin or P2-like myophage tail fibers [13, 16, 17]. The capacity to alter strain specificity by modification of this region implies that these tail fiber fragments must contain receptor adhesin and specificity domain(s).

To date, two myophage tail fiber structures have been reported, T4 gp36 (pdb: 2XGF) and AP22 gp53 (pdb: 4MTM). There is no evident sequence or tertiary similarity that suggests common evolution, domain organization, or function of these proteins. The structure of the T4 distal tail fiber tip is a trimeric helical fiber composed of phage proximal “collar”, medial “needle”, and distal “head” domains [10]. The collar is globular and shares structural homology to a region of the T4 gp12 short tail fiber. The needle is a highly compact trimeric alpha helix with approximately 3 turns and several H-x-H metal-binding sites occupied by Fe^2+^ [10]. The head mushrooms into a small button at the distal C-terminal foot ~10 Å wider than the needle. Unlike T4, the structure of the AP22 tail fiber is an uncharacterized globular trimer.

## Results

### Bioinformatic analysis of R pyocin and myophage tail fiber sequences

**Fig 1:**
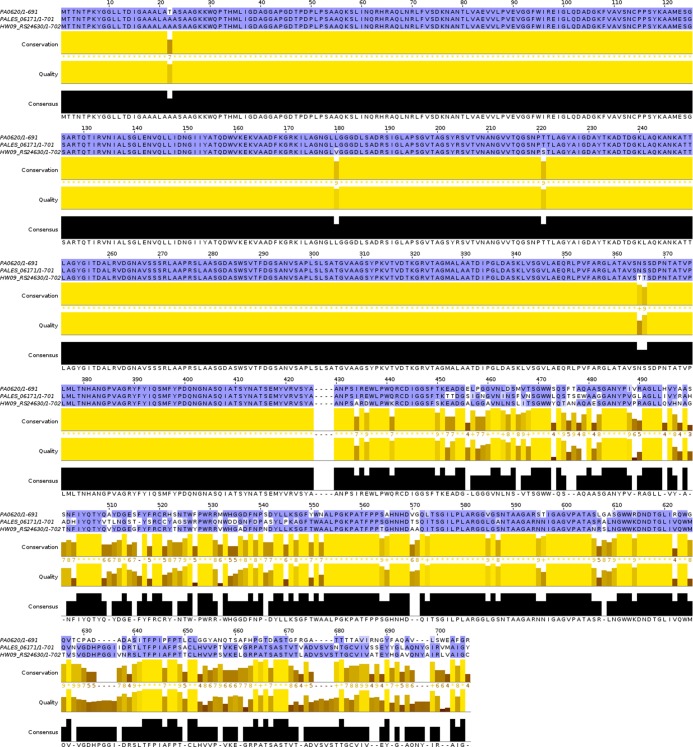
R pyocin tail fibers differ in their NTF. Representative R1, R2, and R5-type pyocin tail fiber sequences were aligned by CLUSTAL Ω and rendered using JALVIEW. Strains aligned: (top) PAO1 –R2, (middle) LESB58 –R1, (bottom) HW09 –R5. Sequence differences between R-subtypes occur in a dense region of polymorphisms and R-subtype independent sequence conservation.

*Pseudomonas* open reading frames (ORFs) with sequence homology to the R2-subtype pyocin tail fiber from *Pseudomonas aeruginosa* strain PAO1 (PA0620) were compiled from the Pseudomonas Genome Database by DIAMOND BLASTP (e cutoff of e < 10^−32^) and aligned by CLUSTAL Ω [18, 19]. Phylogenic assessment revealed that homologous ORFs cluster into three clades that correspond to R-subtype (S1 Fig). Given the strength of these alignments (e < 10^−100^, bitscore > 10^3^, identity > 81%), we annotated putative ORFs as probable R1, R2, or R5-subtype pyocin tail fibers. Representative sequences for R1, R2, and R5-subtype tail fibers were aligned by CLUSTAL Ω and visualized in JALVIEW (Fig 1) [20]. From this alignment, we concluded that R pyocin tail fibers are composed of two regions (numbering relative to native PA0620): a phage proximal region of high conservation (M1-A425) and a distal region of lower conservation (A426-R691). The phage proximal region includes the N-terminal DUF3751 collar-tail domain (M1-A166) and is well conserved between R1 and R2-subtype tail fibers. In the DUF3751 domain of all strains, except *P*. *aeruginosa* isolates Hw09, YQ19, and EG09, R5-subtype tail fibers contain SNPs (D46A, L51I, S53A, A55T, K57T, S58K, H46Y, and A78I) relative to R1 and R2-subtype tail fiber sequences. The remainder of the phage proximal region extending from the DUF3751 domain in the C-terminal direction is highly conserved in all R pyocin tail fibers with only three R5-subtype specific polymorphisms (T221S, N365T, and S366T). Unlike the phage proximal region, the distal tail fiber region contains numerous interspersed segments of variation and conservation, including several INDELS. The density of localized polymorphisms suggests that the distal region is undergoing rapid evolution consistent with the role of a target strain specific adhesin and is, therefore, the focus of our investigation.

In order to further characterize the tail fiber proteins of R pyocins, we investigated sequence similarities of the tail fiber proteins to known myophage ORFs. A pBLAST query against myophage genomes (taxid:10662) in the NCBI non-redundant database returned 32 ORFs with significant homology to the *P*. *aeruginosa* R2-type tail fiber (e score cutoff of e < 10^−20^; S2 Fig). All but four of these ORFs aligned exclusively to the N-terminal DUF3751 domain. Unlike other myophages, the aligned region corresponding to tail fiber ORFs from *Ralstonia* phages RSA-1 (YP_001165272.1), RSY1 (YP_009067102.1), and *Salmonella* phage FSL SP-004 (YP_008239575.1), extended downstream from the DUF3751 domain in the C-terminal direction, terminating at R2-type tail fiber residues L416, D413, and G336, respectively. Only P2-like *Pseudomonas* phage ɸCTX p22 (NP_490619.1) was found to be homologous to the entire R2-type tail fiber (68% identities, 80% positives, and e = 10^−148^; S3 Fig). The similarity of a complete tail fiber sequence was consistent with a growing body of evidence that R pyocins evolved from a temperate ɸCTX-like myophage infection [4, 14, 21].

### Cloning, expression, and purification of N-terminally truncated tail fiber constructs

A region of the R2-subtype pyocin tail fiber of *P*. *aeruginosa*, PAO1, spanning the distal region (R2-NTF; 32.2 kDa) was successfully cloned, recombinantly expressed with an N-terminal his-tag, and purified by Ni^2+^ immobilized metal affinity chromatography (Ni-IMAC). We were unable to produce soluble yields with a corresponding construct of R1-subtype tail fiber from *P*. *aeruginosa*, LESB58. Therefore, we examined additional N-terminal truncations of the R1-subtype distal region. Of constructs tested, only one produced a soluble yield sufficient for crystallization trials (R1-NTF; 16.18 kDa). For both the R2-NTF and R1-NTF, co-expression of respective tail fiber chaperones (PLES06181 and PA0621) was required for obtaining soluble protein yields in excess of 5 mg/mL. When co-expressed, tail fiber chaperones co-purified with their respective his-tagged tail fiber constructs during Ni-IMAC purification. In several trials, denaturation and refolding steps were incorporated to dissociate the complex. Following Ni-IMAC or refolding, fractions containing either construct were purified by size exclusion chromatography (SEC). Comparison of SEC peak elution volumes to those of known SEC standards revealed that the oligomeric state of the R2-NTF was a trimer and that of the R1-NTF was both a dimer and a dimer of trimers (S4 Fig). SEC fractions were pooled, concentrated to between 5–10 mg/mL, and analyzed for purity by SDS-PAGE (S5 Fig). SDS-PAGE reveals that the proteins are >95% pure, with peaks corresponding to predicted masses.

## The R2-NTF is sufficient to bind *P*. *aeruginosa* according to R2-type pyocin sensitivity

**Fig 2:**
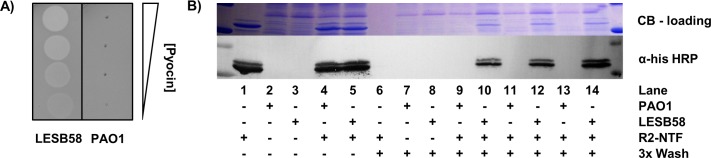
The R2-NTF is capable of target strain specific adhesion. (A) LB agar overlay of LESB58 (left) and PAO1 (right) with spotted R2-type pyocin preparations (PAO1) serially diluted from top to bottom (1:10). (B) (top) Coomassie blue (CB) stained SDS-PAGE gel demonstrating equal loading of cellular protein for the corresponding anti-his blot. (bottom) Anti-his HRP western blot revealing the binding profile of his-tagged R2-NTF to target cells in M9 media. Lanes 9–14 are independent replicates that demonstrate binding of the R2-NTF to R2-type sensitive strain LESB58 (lanes: 10, 12, 14), but not the R2-type insensitive strain, PAO1 (lanes: 9, 11, 13).

R2-type pyocins from *P*. *aeruginosa* strain PAO1 were purified and tested for bacteriocidal activity against known R1 producing strain, LESB58, by agar overlay spotting assay. As anticipated, we found that strain PAO1 was resistant to R2-type pyocin treatment and strain LESB58, on the other hand, was highly sensitive to R2-type pyocins (Fig 2A). To determine if the R2-NTF is sufficient to bind target cells according to the observed R-subtype pyocin sensitivity pattern, R2-NTF was incubated with strain PAO1 or LESB58. Following several rounds of centrifugation and washing to remove unbound R2-NTF, cells were lysed and protein samples normalized for total bacterial protein by SDS-PAGE. We observed (n = 3) in an anti-his western blot that the R2-NTF was specifically bound to LESB58, but not PAO1, confirming that the region of dense polymorphism encapsulated by the R2-NTF defines R-subtype specificity (Fig 2B).

### Structure of tail fiber proteins

**Fig 3:**
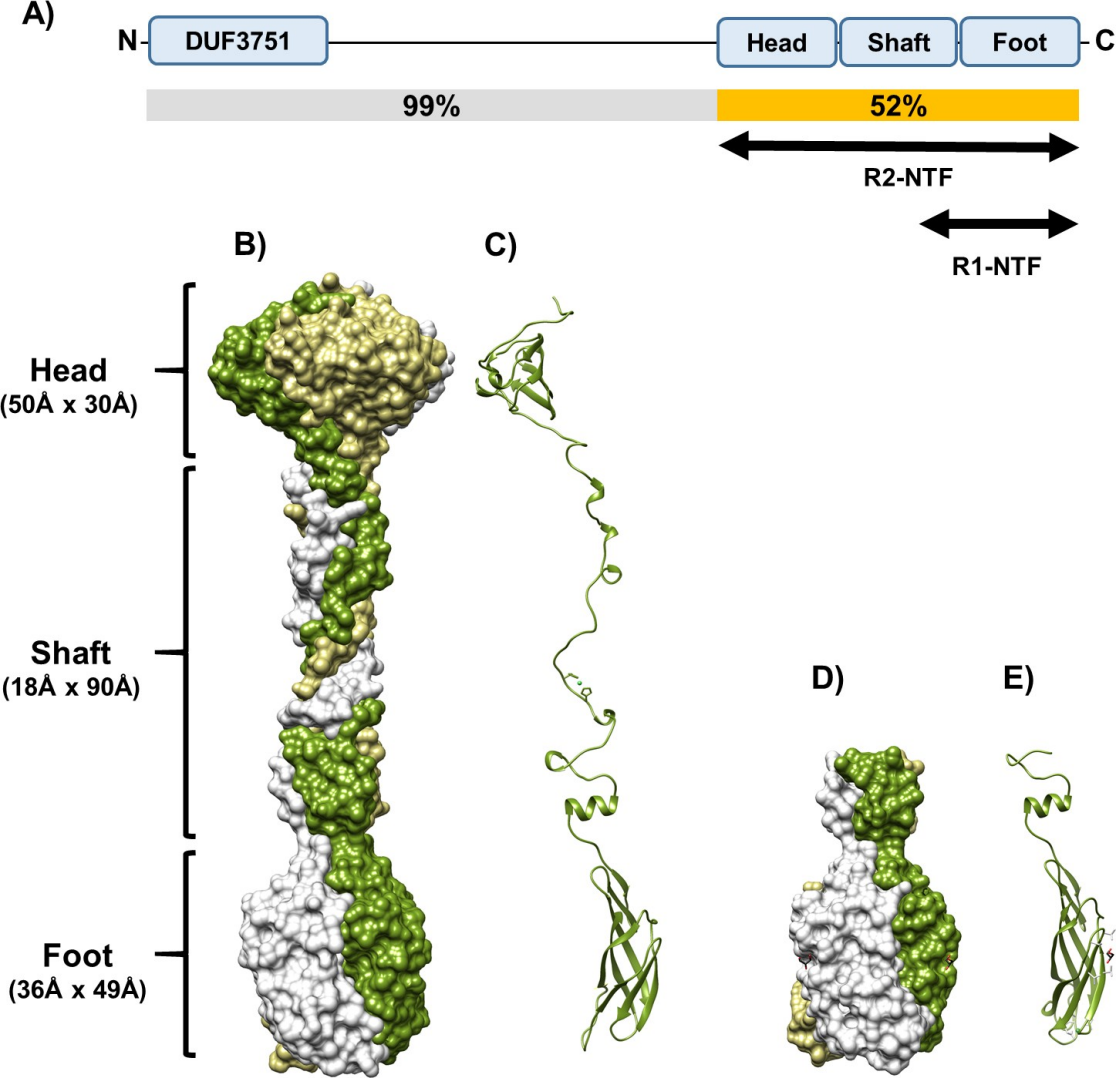
Structure of TFPs are trimeric, helical, and composed of three domains. (A) Cartoon of R1 and R2-NTF constructs highlighting their relative location in the tail fiber and associated domains. Sequence similarity between R1 and R2-subtype tail fibers by region is also displayed (B) Trimeric R2-NTF structure surface rendering: chain A (green) wraps around chain B (beige) and chain C (light grey) in a relaxed helix. (C) R2-NTF single chain ribbon diagram. (D) Trimeric R1-NTF surface rendering showing three chains analogous to the chains of the R2-NTF. (E) R1-NTF single chain ribbon diagram.

We determined the crystal structures of the R1-NTF and R2-NTF to investigate the structural basis of how R pyocin tail fibers recognize their target bacterial strain LPS receptors. Crystals of the two truncated recombinant proteins were produced using the hanging-drop vapor diffusion method [22]. Crystal structures of the R2-NTF were solved by selenium single anomalous dispersion (Se-SAD) and molecular replacement (MR) using the Se-SAD structure as a search model. The R1-NTF structure was solved using molecular replacement with a truncated R2-NTF search model (residues I572 to R691) (Table 1). The packing of these proteins in their respective unit cells showed a clear trimeric arrangement formed by crystallographic symmetry. A trimeric oligomeric state was similarly confirmed by *in silico* analysis with PISA and analytical gel filtration of the biological assembly (S4 Fig). The trimers have approximate dimensions 169 Å x 50 Å x 50 Å (R2-NTF) and 66 Å x 37 Å x 37 Å (R1-NTF). The overall structure of the trimeric R2-NTF is a barbell-like protein, with a three-domain organization consisting of a “head”, medial “shaft”, and “foot”. The head (G443-M525) and foot domains (P598-R691) are globular and connected by an intertwined, helical, and fibrous-looking shaft (W529-V597) (Fig 3). In R1-NTF structures, only the complete foot (V596-Y701) and partial shaft (L580-G595) domains are present.

**Table 1:** Crystallographic data collection and refinement statistics.

Description		Sel-met R2-NTF	R2-NTF	R1-NTF
PDB ID		(6CT8)	(6CU2)	(6CXB)
Data collection	Space group	P6_3_22	P6_3_22	I2_1_3
	Cell dimensions			
	a, b, c (Å)	59.93, 59.93, 396.63	59.87, 59.87, 398.05	102.18, 102.18, 102.18
	α, β, γ (^o^)	90.00, 90.00, 120.00	90.00, 90.00, 120.00	90.00, 90.00, 90.00
	Resolution (Å)^a^	45.98–2.62 (2.67–2.62)	39.80–2.58 (2.62–2.58)	41.71–1.70 (1.73–1.70)
	No. reflections^b^	22500 (13375)	14554	21677
	*R*_merge_ (%)^a^	0.06 (0.12)	0.24 (0.76)	0.07 (0.92)
	*<I>*/σ_*I*_ ^a^	42.9 (18.7)	17.8 (1.7)	30.26 (1.7)
	Redundancy ^a^	7.7 (6.0)	17.2 (5.5)	9.4 (3.6)
	Completeness (%)^a^	93.73 (97.5)	99.45 (98.3)	99.14 (97.6)
	CC_1/2_ ^a^	0.97 (0.93)	0.93 (0.62)	0.93 (0.62)
Refinement	Resolution (Å)	45.98–2.62	39.80–2.58	41.71–1.70
	No. reflections	22500	14554	21677
	*R*_work_/*R*_free_ (%)	0.179/0.234	0.194/0.229	0.159/0.177
	*R*_free_ (test set, %)	10.02	10.0	10.04
	Wilson B-factor (Å^2^)	25.3	35.0	13.0
	Bulk solvent k_sol_(e/Å^3^), B_sol_(Å^2^)	0.38, 33.4	0.32, 30.0	0.36, 44.2
	Fo, Fc correlation	0.92	0.93	0.96
	No. atoms	1982	2053	1050
	No. protein atoms	1889	1888	890
	No. solvent atoms	92	163	153
	No. ligands	1	2	2
	RMSD bonds	0.007	0.009	0.005
	RMSD angles	0.843	0.85	0.788
	Average B, all atoms (Å^2^)	21	32	17
	Sidechain outliers (%)	2.7	0.5	0
	Ramachandran outliers (%)	0	0	0

Root mean squared deviation (RMSD) bond length, RMSD bond angle deviations from ideal stereochemistry, sidechain outliers, and Ramachandran outliers calculated by PHENIX validation software. Other statistics calculated by XTRIAGE or XDS as part of the associated deposition validation reports for PDBs 6CT8, 6CU2, and 6CXB.

(a) Highest resolution shell in parentheses.

(b) Number of total reflections for merged anomalous or merged non-anomalous datasets. The number of reflections for merged non-anomalous data is in parentheses.

#### Head domain of the R2-NTF

**Fig 4:**
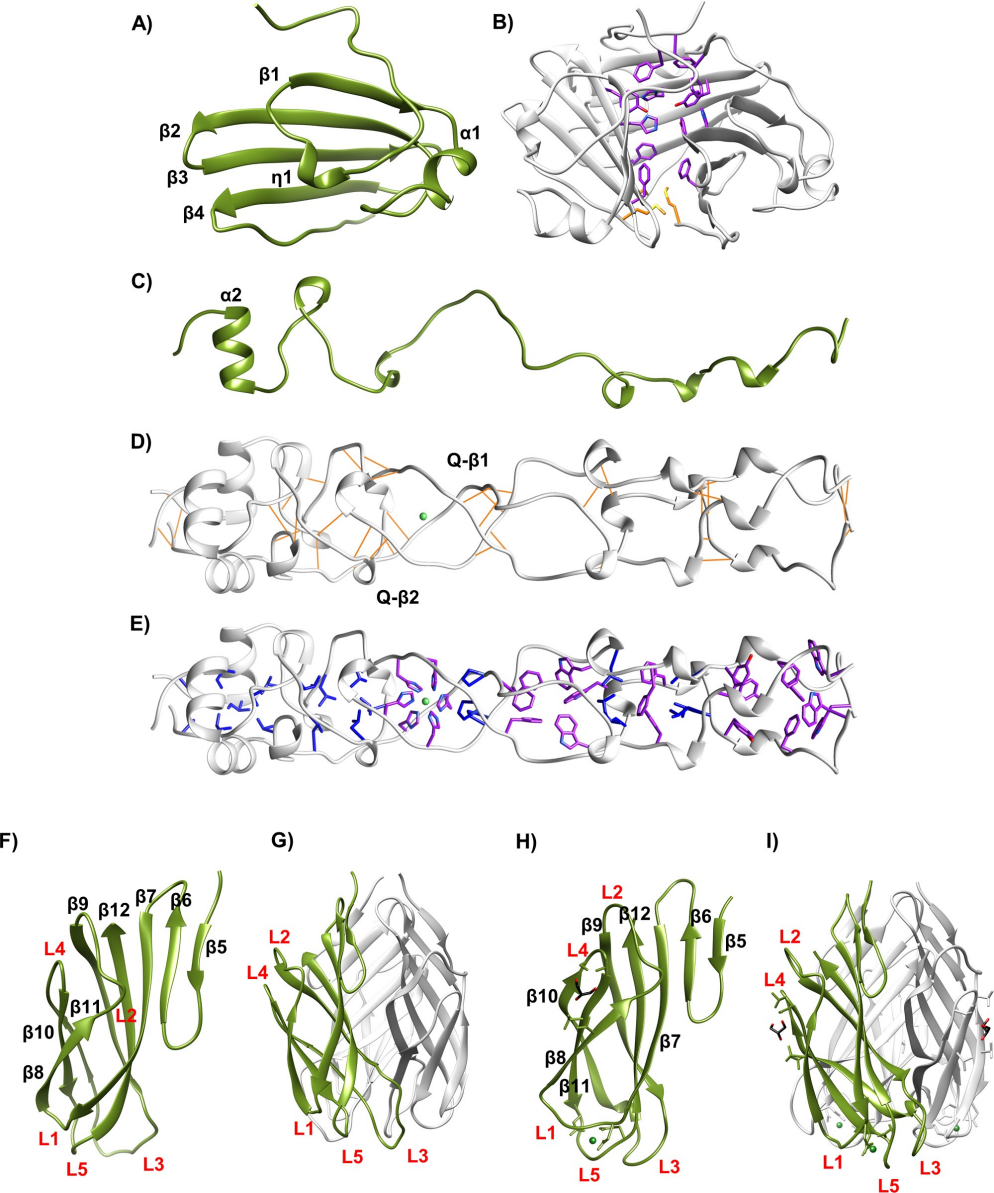
Domain features. (A) R2-NTF head domain, monomeric ribbon diagram with features highlighted. (B) Trimeric head domain–side view. Aromatic interface residues (purple) and tri-methionine cluster (yellow) visible. (C) R2-NTF shaft domain, monomeric ribbon diagram with features highlighted. (D) Trimeric shaft domain–side view with H-x-H site coordinating a divalent cation (green), aromatic interface residues (purple), and hydrophobic residues (blue) visible. (E) Intra-chain hydrogen bonding (gold) and regions of β-like structure highlighted. (F) R2-NTF foot domain, monomeric ribbon diagram with sheet and loop designations (red text) highlighted. (G) Trimeric R2-NTF foot domain with monomer (green), other chains (light grey), and loops (red text) highlighted. (H) R1-NTF foot domain ribbon diagram with features highlighted. (I) R1-NTF foot domain trimer with monomer (green), other chains (light grey), and loops (red text) highlighted.

The head domain is composed of three mixed, anti-parallel β-sheets. Each sheet consists of 4 β-strands (β1-β4). The first strand (β1) is preceded by a short 3_10_ helix (η1), and connected to the remaining β-strands by an additional short helix (α1, T473-A477) and loop (S478-A487). The remaining 3 strands (β2-β4) have a classic, anti-parallel meandering β-sheet motif (Fig 4A). At the center of this globular domain, twelve residues with aromatic rings (F445, H491, Y493, and F512) make π-π interactions at the trimeric interface (Fig 4B). There are inter-chain and intra-chain π-π interactions, including an inter-chain, edge-to-face π-π (H491 to neighboring Y493) and intra-chain interactions parallel to the displaced π-π (F445 to Y493 and H491).

#### Shaft domain

The twisted shaft, which connects the head and foot domains of the R2-NTF, consists of amino acids W529-V597 from each helically intertwined subunit (Fig 3B). The shaft has a rod-like shape with an exterior diameter of 18 Å as measured from the Cα of residue S591 to A587, and a length of 90 Å, as measured from the Cα of residue W529 to the same atom of V597. At the C-terminus of the shaft domain, a single helix (A586-I593) from each subunit forms an equilateral triangle if viewed along the axis of symmetry (Fig 4C). Although other highly organized secondary structure is not evident in any single chain, the shaft trimer contains regions of inter-chain hydrogen bonding, primarily in two short intra-strand quasi-β and α helix backbone interactions (Fig 4D). Similar to the head domain, aromatic and hydrophobic residues line the trimeric interface (Fig 4E). The shaft domain has 8 aromatic (W529, F534, Y539, F545, W547, F557, H562, and H564) and 8 hydrophobic (L540, L550, P559, L569, L574, V581, I593, and A595) residues at the oligomeric interface. Of these, H562 and H564 form an H-x-H motif in octahedral coordination to a large divalent cation of ambiguous identity. In our R2-NTF Se-SAD solution, refinement of log-likelihood gain (LLG) maps revealed a peak at this location differing in displacement from Se-Met, but consistent with predicted scattering from iron series elements (i.e. Fe^2+^, Ni^2+^, or Zn^2+^). ICP-MS confirmed that these metals are associated with the R2-NTF (S6 Fig). However, synchrotron fluorescence x-ray scans were unable to detect a strong peak for any single metal. Although we are currently unable to conclude which metal is present at this site, R2-NTF structures were deposited with Ni^2+^ bound at this position. Not surprisingly, waters are not observed lining the shaft interior.

Like the corresponding region in the R2-NTF shaft domain, the R1-NTF partial shaft domain (L580-V596) is a twisted trimer that contains a single helix (A585-I592). This 17 amino acid region has a Cα RMSD of 0.48 Å compared to the analogous region of the R2-NTF shaft and is 76% identical to the R2-NTF shaft by sequence alignment.

#### Foot domain of the R1-NTF and R2-NTF

**Fig 5:**
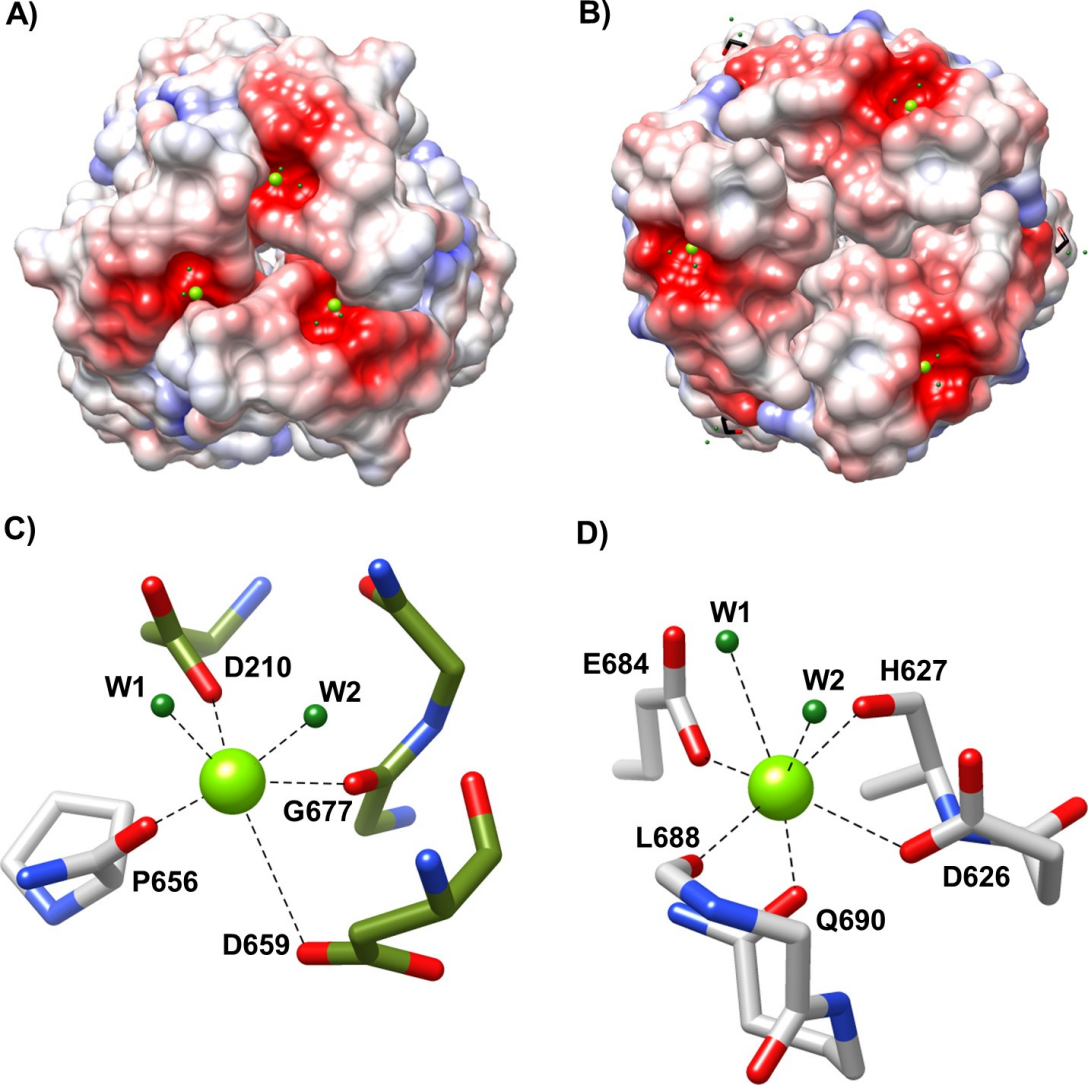
Charged metal binding pocket differs between R-subtype. Both R1 and R2-subtype pyocins have a metal binding pocket in their foot domain. (A) Coulombic map of the R2-NTF foot domain showing a bound small divalent cation of unknown identity (green). (B) Coulombic map of the R1-NTF foot domain showing a bound small divalent cation of unknown identity (green). Both R1 and R2-subtype coulombic maps have a scale of -10 (red) to +10 (blue) kcal/e(mol) charge density. (C) R2-NTF metal binding site at the distal juncture between subunits (green and grey). (D) R1-NTF metal binding site with interactions from a single subunit distal loop network (grey).

The distal foot domain is present in both R1-NTF and R2-NTF structures and contains a conserved β-sandwich jellyroll fold composed of eight anti-parallel β-strands per chain. In each monomer, the first two β-strands (β5-β6) form a meandering, anti-parallel β-sheet. The remaining β-strands (β7-β12) generate a curled jellyroll fold. Connecting these strands are three distal and two proximal loops (L1-L5). L2 and L4 interact significantly and face in the baseplate proximal direction. L1, L3, and L5 are oriented toward the distal extreme of the foot domain (Fig 4F–4I). Between R1 and R2-subtype foot domain structures, backbone positions are highly conserved (Cα RMSD of 1.4 Å) with the exception of the distal loop network (Cα RMSD for L1 of 2.6 Å; L3 of 3.1 Å; L5 of 4.0 Å). Electrostatic surface mapping reveals that the distal loop network forms a negatively charged cavity in both R-subtypes (Fig 5A and 5B). In R2-subtype tail fibers, this cavity is located at the oligomeric interface formed by interactions between L1 and L5 from the same chain and L5 from the adjacent chain, and contains a small divalent cation coordinating two adjacent waters, D628, D659, P656, and G677 (Fig 5C). Similarly, the R1-type tail fiber foot domain has an analogous charged groove located ~9 Å away from the charged cavity of R2-subtype tail fibers. However, unlike R2-subtype tail fibers, it is formed from a single chain with contributing interactions from L1 and L5. It also binds a small divalent cation that is coordinated to R1-NTF residues E684, H627, D626, Q690, L688, and two waters (Fig 5D). The geometry of metal binding in both cases is octahedral.

### Surface polymorphisms reveal putative LPS binding sites

**Fig 6:**
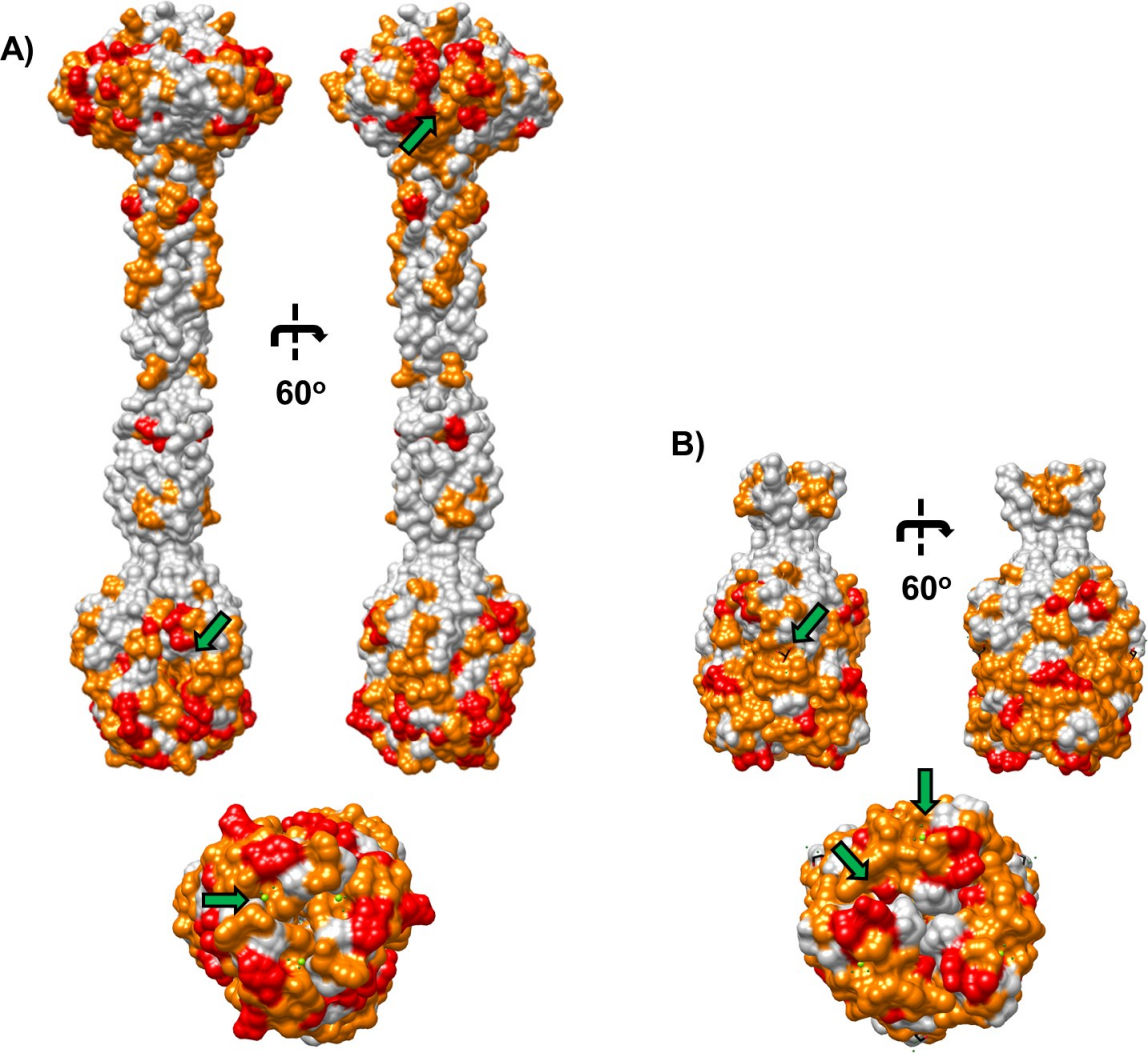
R-subtype polymorphism surface map. Surface rendering of R1 and R2-NTF structures with solvent accessible R-subtype polymorphism (SARP) associated residues highlighted. SARP residue patches in regions of negative curvature reveal putative receptor binding interaction sites (green arrows). (A) R2-NTF structure with surface residues different between all R-subtype pyocin tail fibers (red) and residues different only between R1 and R2-subtype pyocin tail fibers (orange) are highlighted; (top) Lateral view rotated 60^o^, (bottom) Distal view along central axis. (B) R1-NTF structure with surface residues different between all R pyocin tail fiber subtypes (red) and residues different only between R1 and R2-subtype pyocin tail fibers (orange) are highlighted; (top) Lateral view rotated 60^o^, (bottom) Distal view along central axis.

In order to identify potential LPS binding pockets in R pyocin tail fibers, residues corresponding to solvent accessible R-subtype polymorphisms (SARPs) were highlighted in surface models of R1-NTF and R2-NTF structures (Fig 6). In our R2-NTF model, it is evident that polymorphic clusters are located primarily in the head or foot domains. In the head domain, a patch of SARP residues varying between all R-subtypes lines a region of negative curvature at the subunit interface and is flanked by several SARP residues that only differ between R1 and R2-subtype tail fibers. In the foot domains of both tail fibers, a large patch of SARP residues extends over almost the entirely of the foot surface. Interestingly, the distal loop network, which forms the charged metal binding sites and is involved in binding sugars from structurally related jellyroll fold containing adhesins, is composed of SARPs that differ primarily between R1 and R2-subtype tail fibers. Likewise, the R1-subtype specific glycerol binding site (described below) is composed of SARP residues that differ exclusively between R1 and R2-type tail fibers.

**Fig 7:**
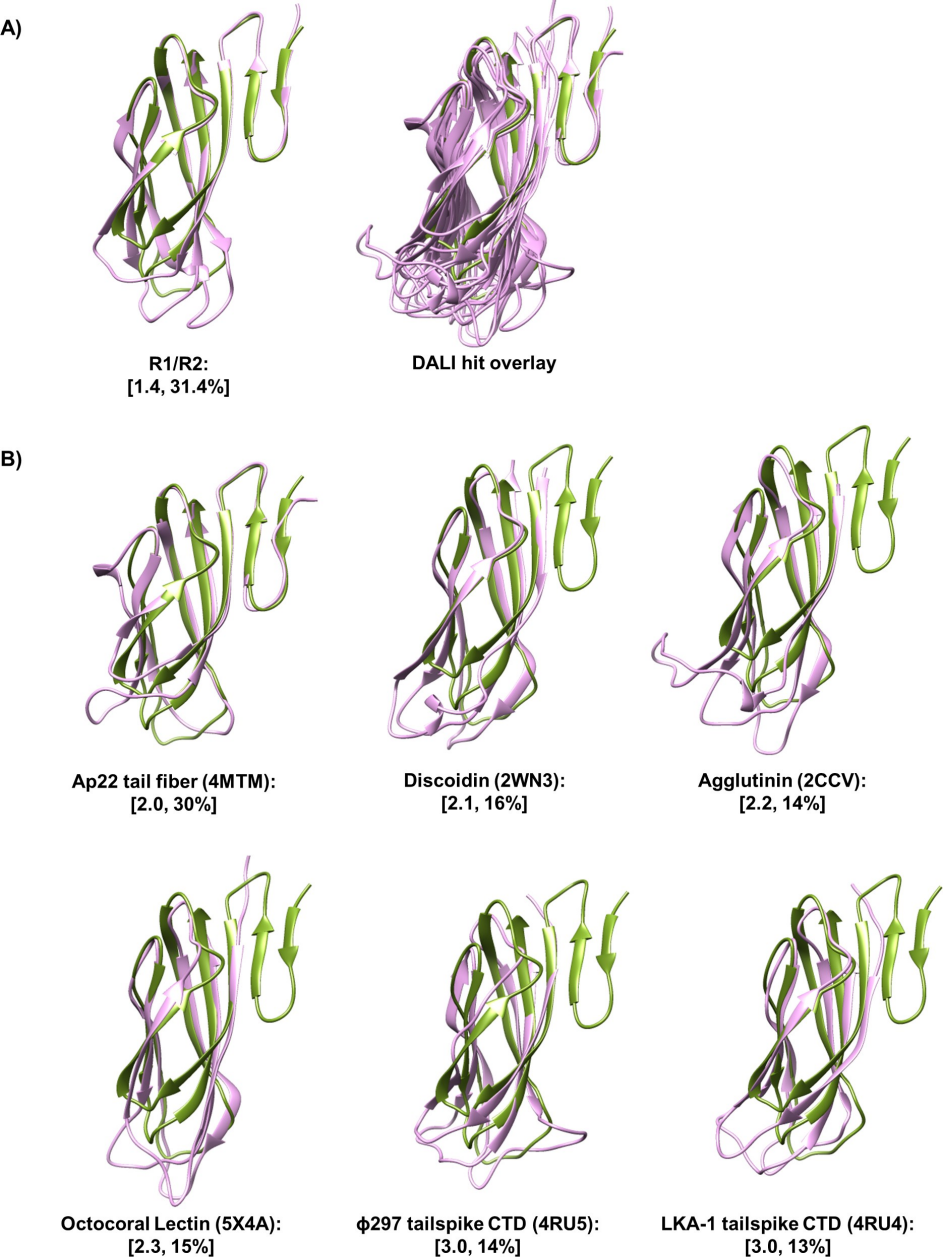
Foot domain is similar to jelly roll fold containing adhesins. Superimposition of DALI hits onto the R2-subtype tail fiber foot domain reveals a conserved lectin jellyroll fold with a variable distal loop network. (A) (left) R1-NTF foot monomer (purple) superimposed onto R2-NTF foot monomer (green); (right) DALI hit structures (purple) superimposed over the aligned region onto the R2-NTF foot monomer (green). (B) DALI hit structures (purple) individually superimposed onto the R2-NTF foot monomer (green). Values in brackets correspond to Cα RMSD and % identity of each structure relative to the R2-NTF foot domain. PDB accession codes are displayed in parenthesis.

With completed structures of the tail fiber proteins, we performed a structural similarity search using the foot domain to known structures in the PDB, as implemented with DALI software [23]. DALI indicated tertiary similarity of the foot domain (Z > 6.5 and identity > 12%) to a region of putative myophage tail fiber AP22 gp53, the C-terminal domain of podophage tail spikes (LKA-1 and ɸ297), and several polysaccharide binding adhesins (agglutinin, discoidin I/II, and octocoral lectin SSL-2) (S1 File). It is evident that over the aligned region, these proteins share a common jellyroll fold with a distal loop network analogous to that of R pyocin tail fiber foot domain loops L1, L3, and L5. Not surprisingly, the distal loop network of these proteins is highly variable, with vastly different loop lengths (Table 2) and conformations (Fig 7). Superimposition of DALI hits onto R-subtype pyocin foot domains with CHIMERA revealed a high degree of similarity in the conservation of backbone β-strands and loops L2 and L4 (Cα RMSD of 2.0 Å), but not L1 (Cα RMSD of 3.7 Å), L3 (Cα RMSD of 3.2 Å), or L5 (Cα RMSD of 4.0 Å).

**Table 2:** Distal loop network of foot domain containing proteins.

	Aligned residues	L1	L3	L5
R1-NTF (foot)	V596-Y701	G625-I632	E655-V666	E684-N691
R2-NTF (foot)	P598-R691	P626-A629	S652-A660	Y678-Q681
AP22 gp53 (4MTM)	N174-Y271	T202-T204	N228-I233	Q253-G261
Discoidin I (2WN3)	S156-E253	G168-V182	I205-I216	T234-S242
Agglutinin (2CCV)	R1-E99	G10-L31	Q53-V64	T82-S90
LKA-1 gp49 (4RU4)	P676-G765	T689-V697	D719-G728	T746-T757
ɸ297 gp27 (4RU5)	T650-S742	T662-V669	S692-P706	A724-S734
Octocoral lectin (5X4A)	R1-D94	R16-S26	M48-V59	S77-N85
Overall RMSD (Cα)	2.4	3.7	3.2	4.0

### R1-NTF glycerol binding pocket highlights putative R-subtype specific LPS interaction site

**Fig 8:**
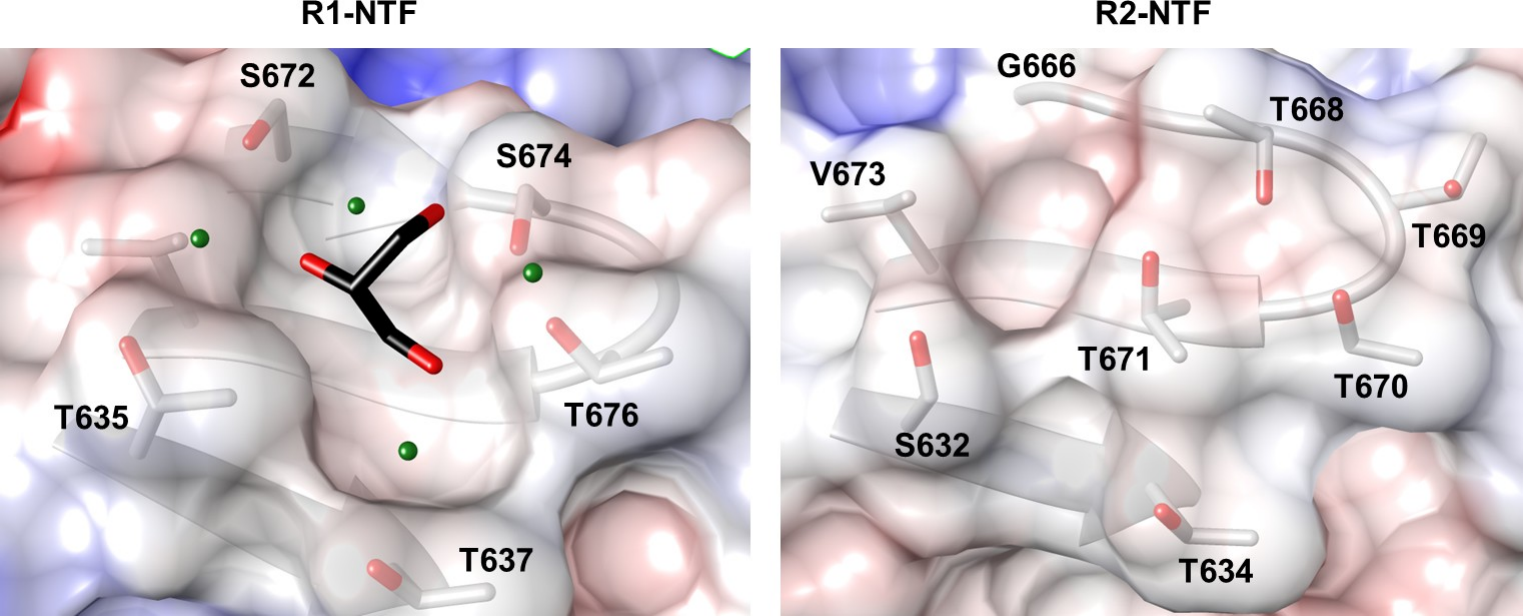
R1-subtype foot domain glycerol binding site is not present in R2-subtype tail fiber NTF. Glycerol binding pocket with all residues within 6 Å of the ligand highlighted. (left) R1-NTF surface coulombic map with glycerol ligand (black, colored by element); (right) Surface coulombic map of analogous aligned region in the R2-NTF foot. Comparison of the binding pocket between the R1-NTF and R2-NTF foot domains suggests feasible binding of glycerol to the R1-NTF only. Glycerol is capable of binding carbohydrate pockets in conformations similar to those used by sugar ligands. The presence of glycerol could indicate that this site is involved in R1-type specific tail fiber interactions with target strain LPS.

Glycerol, a carbohydrate backbone fragment, is often bound to sugar binding sites in protein structures, and has been previously used as a surrogate ligand for identification of carbohydrate interaction pockets [24]. Although glycerol is abundant in R1-NTF and R2-NTF protein preparations, only the R1-NTF structure contains a high occupancy glycerol at a site formed by residues T635-F638 (β8) and S672-I680 (β10, L4, and β11), and located ~25 Å away from the distal loop network. Unlike the distal tip of the loop regions, the R1-NTF glycerol pocket is uncharged and contains coordinating waters. This pocket is composed of residues S672, S674, T676, T637, and T635 (Fig 8A). Investigation of the corresponding region in the R2-NTF foot domain (38% identical) reveals the existence of a much smaller pocket composed of residues S214-F217 (β8) and G248-I256 (β10, L4, and β11), that without significant conformational changes would sterically inhibit glycerol or LPS component sugar binding (Fig 8B).

## Discussion

In our investigation, we successfully elucidated the structures of R1 and R2-subtype pyocin tail fibers, allowing us to describe the R-subtype variable region, its domain composition, and cell binding function. These structures are in agreement with the recently reported structures of Buth et al (2018) that contain different fragments of R1 and R2-subtype tail fibers than described in this report. The R-subtype variable region investigated here contains 99% of the polymorphic variation between R-subtype tail fibers. Because there is no known specific conserved function of tail fibers beyond binding host or target cells, we hypothesized that sequence differences between R-subtype tail fibers would ostensibly correspond to residues involved in R-subtype selective binding and most of the residues involved in these interactions would belong to the variable region. We, therefore, propose that the head or foot domains found within this variable region contain putative LPS binding motifs. Our structures suggest that the discoidin-like “foot” is responsible for strain specificity and core LPS binding. In our model, R pyocin specificity by means of core LPS sugar binding is accomplished by host sugar interactions with the distal loop network of the foot domain. Supporting our assertion is evidence that structurally similar distal loop regions of discoidin and related lectins interact with polysaccharides (see below). In both our R1 and R2-subtype tail fiber structures, the distal loop network contains charged metal binding sites atypical in characterized discoidin-like lectins. However, unrelated lectin structures contain charged metal binding sites in which Ca^2+^ interactions stabilize residues of a sugar binding groove indirectly through orienting residues involved in sugar binding (Concavalin A, PDB:1I3H) and, in which Ca^2+^ is directly involved in coordinating hydroxyl interactions of the target carbohydrate (Rat mannose binding C-type lectin, PDB:2SMB) [25]. Therefore, we have reason to believe that metal interactions are involved in mediating LPS binding of R pyocin tail fibers.

### The foot is a discoidin-like lectin domain

As revealed by DALI, the adhesins discoidin I/II, agglutinin, and octocoral lectin share significant structural homology with the R pyocin tail fiber foot domain. These lectins contain polysaccharide binding sites in their distal loop network capable of accommodating N-acetyl-galactosamine and other carbohydrates [26, 27]. However, there is little obvious commonality between the sugar binding sites of these adhesins other than their use of a variable distal loop network to bind sugars moieties. In discoidin-like lectins and R pyocin tail fibers, a jellyroll fold provides a scaffold for contextual alignment of three loops, which we have designated the distal loop network. Like the variable loop system of antibodies, these loops are capable of forming a wide array of context-specific binding sites. In our investigation of the R pyocin tail fiber foot domain, we revealed that the foot domain distal loop network is highly variable in both sequence and structure between R1 and R2-subtype tail fibers. Given that the distal loop network is the sugar binding site of discoidin-like lectins and that the remainder of the foot domain structure has low (< 1.5 Cα RMSD) positional deviation between R-subtype, we posit that the foot domain is an R-subtype specific adhesin domain.

### Similarity of R pyocin tail fiber domains to known phage structures

To the best of our knowledge, the head and shaft domains of the R2-NTF are structurally distinct from any other proteins in the PDB with the exception of a report concurrent with this submission by Buth et al (2018) [28]. The R2-NTF shaft domain, however, shares characteristics with the T4 distal tail fiber shaft. Like T4, the R2-NTF shaft contains a H-x-H motif that forms an octahedral metal coordination site at the trimeric interface, and is an intertwined helix lacking canonical intra-chain secondary structure. This H-x-H motif is conserved in R pyocin tail fiber sequences and in the tail fiber sequence of phage ɸCTX. Unlike the R2-NTF structure, which contains a single H-x-H metal binding site, the T4 shaft contains several H-x-H sites within a longer, tighter helix. Therefore, we hypothesize that a fibrous region of elongated helical character containing one or more H-x-H metal binding site is a common structural feature of tail fibers and, perhaps, serves to stabilize the elongating fiber during trimerization.

Our study reveals that R pyocin foot domains are similar to the myophage AP22 tail fiber (pdb: 4MTM). Both the AP22 tail fiber and R pyocin foot domains are composed of a trimeric discoidin-like fold with divergent positions of L1, L3, and L5 loops. In both the R pyocin and AP22 foot domains, a cavity at the distal trimeric interface is formed by loops L1, L3, and L5. In both R1-NTF and R2-NTF structures, this groove is polar, negatively charged, and coordinates a small divalent cation. Unlike R pyocin tail fibers, the AP22 distal foot cavity is hydrophobic, uncharged, and contains a bound ethanediol ligand. In AP22, a single glycerol is also coordinated to a lateral foot hydrophobic groove at a different position than the bound glycerol in the R1-NTF structure (S7 Fig). Although sharing jellyroll backbone homology, differences in surface features suggest that AP22 and R pyocin tail fibers might bind different host receptors.

Like tail fibers, phage tailspikes attach to a hexagonal baseplate at N-terminal “hinge” regions and are responsible for coordinating interactions with host receptors in such a way as to orient and anchor baseplate machinery for genome injection (S8 Fig). [29]. Similarly, most characterized tailspikes are trimeric and have a long axis of symmetry. Unlike tail fibers, tailspikes are typically composed of small N-terminal or C-terminal domains flanking an elongated β-barrel domain [29, 30]. Several characterized tailspikes, including those of podophages P22, LKA-1, and ɸ297, display endorhamnosidase or capsular exopolysaccharide (EPS) depolymerase activity dependent on binding O-antigen LPS in their β-barrel domains [31–33]. Unlike the P22 tailspike, the C-terminal domains of LKA-1 and ɸ297 tailspikes are discoidin-like folds structurally related to R pyocin foot domains. Although the β-barrel domain of LKA-1 and ɸ297 tailspikes is believed to be the primary site of host LPS binding [33], the presence of a discoidin-like C-terminal domain oriented to make contact with core LPS suggests that this class of tailspike might also be implicated in binding host core LPS.

### Prediction of LPS binding sites from structural data

We elucidated the presence of two large patches of surface residues polymorphic between R-subtypes in the head and foot domains. We hypothesize that at least one of these patches contains sites capable of binding and differentiating between target strain LPS. Assuming that membrane contact is made by the tail fiber at the distal loops of the foot domain, as described above, we believe that the entirety of the head domain is likely restricted to interaction with LPS o-antigen. In the case of rough strains lacking o-antigen, such as LESB58, core LPS interactions are probably restricted to interaction with only a portion of the foot domain [15].

### Key findings and implications

Briefly, our investigation posits several key findings. Specifically, we report that N-terminally truncated R1-subtype and R2-subtype pyocin tail fiber fragment structures include three domains found in a region of variation between R-subtypes. We further find that this region is responsible for strain specific binding. We hypothesize that the likely LPS receptor or specificity domains are found in the “head” and “foot” domains. Our structures also reveal a unique glycerol binding site available in R1-subtype, but not R2-subtype pyocin tail fibers, that putatively indicates the involvement of nearby residues in R1-subtype specific LPS interactions.

Several important avenues of research stem from these findings. Specifically, we believe that analysis of the metal binding requirements of adsorption would further elucidate the specific roll that metals undoubtedly play. It is also reasonable to anticipate that a detailed investigation of head and foot domain mutants and 2D-NMR perturbation analysis upon core or O-antigen LPS binding, would confirm our finding that one or both of these domains are responsible for mediating LPS interactions. Overall, these studies would provide a more comprehensive understanding of tailocin adhesion that is integral to the development of effective phage-based biotics, including modified R pyocins and targeting peptides for drug delivery.

## Materials and methods

### Cloning of recombinant tail fiber and chaperone constructs

Truncation constructs R1-NTF and R2-NTF spanning the distal region were amplified by PCR from genomic DNA of *P*. *aeruginosa* strains LESB58 (PALES_06171; I572-Y701) and PAO1 (PA0620; Y419-R691), respectively. Amplicons were cloned, in frame, into plasmid pMCSG11 LIC sites using ligation independent enzymatic assembly [34]. Resultant plasmids (pMCSG11/R1-NTF and pMCSG11/R2-NTF) were transformed into BL21 (DE3) *E*. *coli* and selected on LB agar supplemented with chloramphenicol (50μg/mL). In order to obtain sufficient soluble expression, the corresponding tail fiber chaperones from LESB58 (PALES_06172) and PAO1 (PA0621) were cloned into pET28-TEV using restriction sites NdeI and XhoI. Chaperone expressing plasmids were co-transformed with plasmid pMCSG11/R1-NTF or pMCSG11/R2-NTF, as appropriate, into BL21 (DE3) *E*. *co*li. Co-transformants were selected on LB agar supplemented with chloramphenicol (25μg/mL) and carbenacillin (100μg/mL). All inserts were sequenced by the Sanger method.

### Expression and purification of R1-NTF and R2-NTF

BL21 (DE3) cells co-transformed with NTF expression and chaperone vectors were grown on an orbital shaker at 37°C until reaching a mid-log OD_600_ of 0.6. Cells were chilled on ice for 30 min prior to induction with 1mM isopropyl 1-thio-β-D-galactopyranoside (IPTG) at 16°C for 16 hours. Induced cells were pelleted by centrifugation. Pellets were stored in 20 mL of Ni-IMAC wash buffer (see below) at -20°C until needed and lysed by a single passage at 20,000 psi using a French press (M-110P; Microfluidics Corporation). Lysates were centrifuged to remove cell debris at 15,000 rpm for 45 minutes at 10°C. NTF-chaperone complexes were purified by nickel immobilized metal affinity chromatography (Ni-IMAC) using an AKTA FPLC (GE Healthcare Lifesciences). A HisTrap FF Ni-IMAC resin column (GE Healthcare) was equilibrated with 50mL of wash buffer (50mM Tris pH 8.0, 150mM NaCl, 10mM imidazole, and 5% glycerol) prior to the addition of 0.45μm filtered lysates. Unbound protein was removed by washing with 500mL of wash buffer. Protein was eluted from the column using 20mL of elution buffer (50mM Tris pH 8.0, 150mM NaCl, 300mM imidazole, and 5% glycerol). Upon elution, proteins were dialyzed in wash buffer lacking imidazole (50mM Tris pH 8.0, 150mM, and 5% glycerol). As needed, NTF-chaperone complex(s) were denatured on column with buffered urea (50mM Tris pH 8.0, 500mM NaCl, 1M Urea, and 10% glycerol) at 25°C. Following denaturation, unfolded R1-NTF or R2-NTF samples were washed with an excess of denaturation buffer to remove unbound chaperone. Samples were refolded by incubating IMAC resin in refolding buffer (50mM Tris pH 8.0, 500mM NaCl, 5% Glycerol). Refolded protein was eluted as described previously. Peak fractions were pooled, dialyzed overnight in dialysis buffer (50mM Tris pH 8.0, 150mM NaCl, 5% glycerol) at 4°C, and further purified by size exclusion chromatography (SEC) on a Sepharose-200 column equilibrated with dialysis buffer. Peaks corresponding to each protein were pooled, concentrated to 4–5 mg/mL, and analyzed for purity by SDS-PAGE (S5 Fig). Sample oligomeric state was determined by SEC on an analytical Sepharose-200 10/300 pre-packed column (GE Healthcare Lifesciences) and verified by PISA [35]. For analytical gel filtration, R1-NTF or R2-NTF samples were injected 3 column volumes following the injection of rehydrated SEC standards (Biorad) or previous protein samples diluted to 1mg/mL in dialysis buffer.

### Selenomethionine replacement of the R2-NTF

For initial solutions, the R2-NTF was labelled with selenomethionine prior to crystallization and anomalous phasing [36]. Plasmids pMCSG11/R2-NTF and pET28TEV/PA0621 were co-transformed into methionine auxotroph B834 (DE3) *E*. *coli* (Novagen), and selected on LB agar. 40mL starter cultures were grown from co-transformants overnight in LB. Cells were separated from media by centrifugation and the liquid supernatant was discarded. Cell pellets were washed in 0.22μm filtered M9 minimal media lacking a carbon source. Thoroughly washed cells were then used to inoculate 2L of 0.22μm filtered M9 minimal media supplemented with L-selenomethionine (60mg/L) and a complete mix of amino acids (60mg/L) lacking L-methionine. Subsequent expression and purification of the R2-NTF was performed identically for selenomethionine derived and non-derived recombinant protein.

### R2-type pyocin purification

*P*. *aeruginosa* PAO1 cultures were grown overnight in LB media incubated at 37°C with agitation. Pyocin production was induced by exposure to germicidal UV for 1min at 5min intervals (n = 6), and grown overnight [2]. Cultures were centrifuged to remove cells and lysis debris from the supernatant containing crude pyocin particles (30 min at 4K rpm). Supernatants were treated with trypsin (5ug/mL) and incubated at 37°C for 2h to inactivate contaminating S-type and F-type pyocins. To remove trypsin, bacteriocidal small molecule compounds, protein fragments, or residual S pyocins from the culture media, supernatants were filtered (0.22μm PES) and buffer exchanged with sterile M9 media (Sigma) using 100kDa cutoff concentrator dialysis (Centricon) for 10 volumes (100mL). To inactivate any residual induced phages, preparations were plated in an open petri dish within a biological safety cabinet and continuously irradiated with germicidal UV for 15min. These crude preparations were filtered to remove any aggregates and analyzed for purity by SDS-PAGE (S5 Fig).

### R2-type pyocin sensitivity assay

Bacteriocidal activity of R2-type pyocin preparations was determined using a modified version of the agar overlay spotting technique [37]. Liquid cultures of strains LESB58 and PAO1 were grown overnight in LB media. Cultures were diluted in fresh LB to an OD_600_ of 0.8 and 100μL of culture was added to 10mL of molten top agar (0.6% LB agar). Inoculated top agar was overlaid onto LB agar plates (18mL) and allowed to dry. R2-type pyocins were serially diluted (1:10 or 1:1) in sterile M9 media, and 3μL aliquots of each dilution were spotted on the dry top agar surface. Once aliquots were fully absorbed, plates were incubated overnight at 37°C prior to observation. For sensitivity testing involving chelators, chelator stocks (pH 7.2) were sterile filtered (0.2μm) and added to both molten primary and top agar to final concentrations reported (0.25μM– 5μM).

### R2-NTF adhesion assays

Overnight LB cultures of strains LESB58 and PAO1 were centrifuged (4K rpm for 10 min) and washed with 0.22μm filtered M9 media (Sigma). Supernatant was removed and replaced with 800μL of fresh M9 media. After resuspending cells, 100μL aliquots were distributed to individual microcentrifuge tubes. Frozen aliquots of purified R2-NTF were buffer exchanged with 50mM Tris 7.0, 150mM NaCl using 10kDa cutoff concentrator dialysis for 10 volumes (20mL) and concentrated to a final concentration of 4mg/mL. 5μL of R2-NTF protein was added to samples, as appropriate, and incubated for 10 min. Samples were then centrifuged at 13K rpm for 15 min and the supernatant containing unbound R2-NTF protein was removed. Cells were washed with 200μL of fresh M9 media, centrifuged, and the supernatant was removed (n = 3). Washed pellets were lysed with 100μL of Bugbuster (EMD Millipore) for 30 min. Lysate concentration was normalized by total bacterial cellular protein as determined by SDS-PAGE. Normalized samples were separated by SDS-PAGE and transferred to an ethanol pre-wetted PVDF membrane by electrolytic transfer at 4°C for 1h in modified transfer buffer lacking methanol (25 mM Tris pH 8.6, 200 mM glycine, 0.1% SDS, and 20% ethanol). Upon completion of transfer, membranes were washed in TBST wash buffer (50mM Tris pH 7.5, 150mM NaCl, and 0.1% Tween 20), and successful transfer was confirmed by reversible staining with Ponceau S dye (Sigma). Membranes were washed with TBST and blocked in TBST containing 5% dry milk powder for 1h. Blocked membranes were transferred to fresh TBST with milk powder and mouse anti-his HRP conjugate antibody (1:5000 dilution, Sigma). Membranes were incubated with antibody for 1h and subsequently washed with excess TBST. Thoroughly washed membranes were decanted and exposed to 10mL of Ultra-TMB developer (Thermo) for 5 min. Developed membranes were washed in 50mL of TBST prior to imaging.

### Crystallization and x-ray diffraction analysis

High-throughput precipitant arrays (Hampton) were screened for suitable crystallization of constructs by sitting and hanging drop vapor diffusion methods [22]. Sitting drops were composed of protein in a 1:1 ratio of precipitant condition to protein (10/6/5 mg/mL) at 16°C. Crystallization hit optimization was performed in both 96-well sitting drop and 24-well hanging drop plates (Hampton) by varying the concentration of protein and volume ratio of precipitant condition to protein in the crystallization drop. Selenomethionine replaced R2-NTF crystals were grown at a 1:1 ratio of protein (6mg/mL) to precipitant (1M Na Acetate, 100mM CAPS, 100 mM LiOAc) and diffracted up to 2.6Å resolution. Non-refolded R2-NTF crystals were grown under the same conditions as selenomethionine replaced crystals, but with a different precipitant (100mM HEPES pH 8.0, 1M Sodium acetate, 0.4mM LDAO) and diffracted up to 2.5 Å resolution. R1-NTF crystals were grown at a 1:1 protein (5 mg/mL) to precipitant (100mM Bicine pH 9.5, 20% PEG2k, 1mM CuSO_4_) ratio and diffracted up to 1.7Å resolution. In both cases, crystals appeared in 3–7 days and were morphologically single or layered hexagonal prisms. Prior to data acquisition, crystals were flash frozen with a cryoprotectant of 30% glycerol and 70% mother liquor and kept at a constant 120K during data collection. Diffraction images were collected on a Riggaku R-axis rotating Cu-anode home source (PDB: 6CU2), at Berkley Advanced Light Source (ALS) beamline 5.0.2 (PDB: 6CXB), and at Argonne National Laboratory Advanced Photon Source (APS) beamline 23ID-D (PDB: 6CT8). The wavelength utilized for synchrotron data collection was 0.98 Å. Reflection data was processed and scaled using HKL2000 [38]. R2-NTF datasets were scaled into space group P6_3_22 and R1-NTF datasets were scaled into space group I2_1_3.

### Structure determination and refinement

The R2-NTF apostructure was determined using single wavelength anomalous dispersion (SAD) of selenomethionine-replaced R2-NTF crystals. Heavy atom sites were identified, phased, and refined from scaled reflections by AUTOSOL [39]. An initial model was generated and refined using AUTOBUILD [39]. A high quality single chain structure was constructed following cycles of manual model building in COOT and refinement with REFINE [39, 40]. High-resolution R2-NTF structures were solved by molecular replacement with PHASER (MR) using the Se-SAD structure as a search model [39]. The R1-NTF structure was solved by PHASER (MR) and built in AUTOBUILD using a portion of the R2-NTF apostructure (V163-A273) as a search model. Iterative model building in COOT and refinement in REFINE was used until structure completion.

### ICP-MS determination of associated metals

Protein samples were concentrator dialyzed (10kDa cutoff) in buffer (20mM Tris 7.2 and 150mM NaCl) for 500 volumes in the presence or absence of 5mM EDTA (pH 7.2). Samples were then digested with nitric acid and hydrogen peroxide in a Milestone UltraWave. Digests were injected, processed, and analyzed on a Perkin Elmer DRC 2 ICP-MS system by the Texas A&M University Trace Element Lab.

### SDS-PAGE analysis

Protein samples were mixed with running buffer, heated to 95°C for 10 min, loaded onto an SDS-PAGE gel (Biorad), and run at 200V. Gels were stained with Coomassie Blue dye for 20 min and destained in water for 4hr before visualization.

## Supporting information

S1 Fig Alignment of Pseudomonas R tail fibers reveals three distinct clusters. Complete genomes from the Pseudomonas Genome Database were queried by DIAMOND BLASTP using the R2-type pyocin tail fiber from PAO1 (PA0620) as a search sequence. Hit ORFs were aligned by CLUSTAL Ω and a BLOSUM62 neighbor-joining tree was generated from the results. The tree shows clustering of pyocin tail fiber sequences into three distinct branches, predominated by R-subtype.
(TIF)

S2 Fig R pyocin tail fiber contains conserved, P2-like myophage N-terminal DUF3751 domain. R2-type pyocin tail fiber sequence from *P*. *aeruginosa*, strain PAO1, was used as the subject for a pBLAST query against NCBI genomic database myophage genomes. Myophage genome hit ORFs were extracted, aligned by CLUSTAL Ω, and the resulting alignment rendered in JALVIEW. Individual sequences are colored from white (low conservation) to dark blue (high conservation). The alignment conservation score (0–9), relative quality of genomic data, and consensus sequence, appear as separate tracks with associated sequence positions.
(TIF)

S3 Fig R pyocin tail fibers are closely related to myophage ɸCTX. JALVIEW rendering of CLUSTAL Ω alignment between the R2-type pyocin tail fiber and ɸCTX contig containing the p22 tail fiber C-terminus. Alignment colored according to the BLOSUM62 convention.
(TIF)

S4 Fig Size exclusion chromatography of R1-NTF and R2-NTF. Analytical SEC reveals oligomeric state(s) of purified R1-NTF and R2-NTF. (A) UV chromatograph of R1-NTF (orange), R2-NTF (green), and SEC S-200 standards (blue dashes) showing peak elution times. (B) Plot of elution volume vs. molecular weight for SEC standards. Exponential regression reveals that peak 1 for the R2-NTF corresponds to a trimeric oligomer, and peak 2 for the R1-NTF corresponds to a dimer of trimers.
(TIF)

S5 Fig SDS-PAGE analysis of R2-pyocin and NTF purity. SDS-PAGE gels stained with Coomassie blue dye. (A) Purified R2-pyocin preparations (20μL); (lane 1) M9 buffer exchanged LB media. (lane 2) M9 buffer exchanged LB media with purified PAO1 LB supernatant. (lane 3) Precision plus protein standards, sizes listed in kDa. (B) Purified NTF samples used in crystallization and cell binding experiments; (lane 1), R2-NTF sample (1μL of 5mg/mL protein). (lane 2) R1-NTF sample (1μL of 5mg/mL protein). (lane 3) Precision plus protein standards, sizes listed in kDa.
(TIF)

S6 Fig ICP-MS reveals several metals are bound to refolded R2-NTF. R2-NTF samples were buffer exchanged and concentrated to 1mg/mL. Fractions containing protein and concentrator flow-through were analyzed by ICP-MS for the presence of divalent cations. Plot of fold change between flow-through and protein fractions (n = 2).
(TIF)

S7 Fig AP22 and R1-NTF differ in binding of ligands. Hydrophobicity (top) or Charge scoring (bottom) of residues in the tail fiber of myophage AP22 (left) and R1-NTF (right). In AP22, ethanolamine and glycerol bind hydrophilic pockets absent in the R1-NTF structure.
(TIF)

S8 Fig Known phage adhesin structures. Structures of tail spikes and fibers compiled from the PDB and rendered in CHIMERA.
(TIF)

S1 File DALI search results. (XLSX)

## Abbreviations

R1-NTF: R1-subtype N-terminally truncated tail fiber fragment
R2-NTF: R2-subtype N-terminally truncated tail fiber fragment
SARPs: solvent accessible R-subtype polymorphisms
