# Supplementary figures and images for: R pyocin tail fiber structure reveals a receptor-binding domain with a lectin fold
Source: PLoS One. 2019 Feb 5;14(2):e0211432. doi: 10.1371/journal.pone.0211432 (PMC6363177; doi:10.1371/journal.pone.0211432)

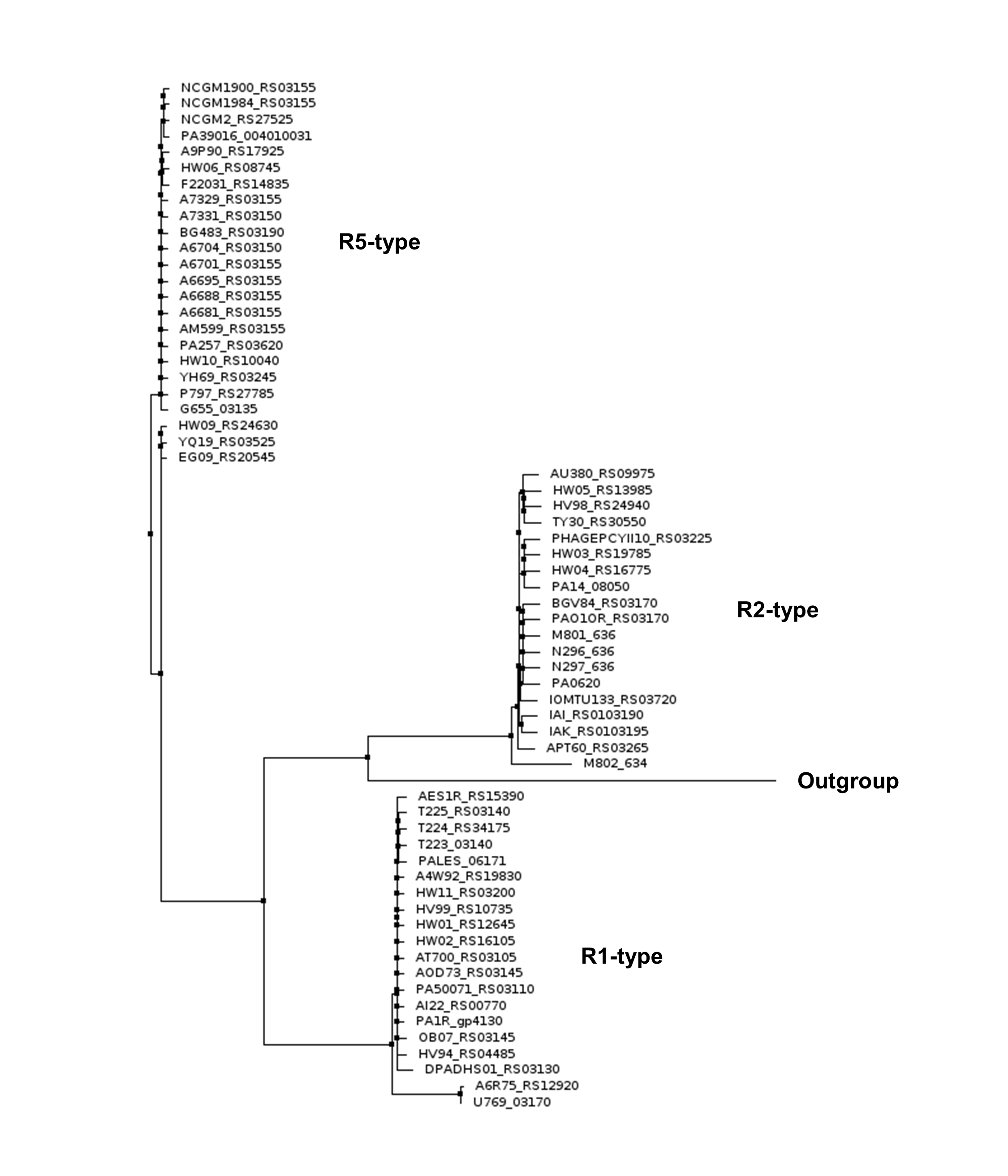

Supplement: S1 Fig — Complete genomes from the Pseudomonas Genome Database were queried by DIAMOND BLASTP using the R2-type pyocin tail fiber from PAO1 (PA0620) as a search sequence. Hit ORFs were aligned by CLUSTAL Ω and a BLOSUM62 neighbor-joining tree was generated from the results. The tree shows clustering of pyocin tail fiber sequences into three distinct branches, predominated by R-subtype. (TIF) [file pone.0211432.s001.tif]

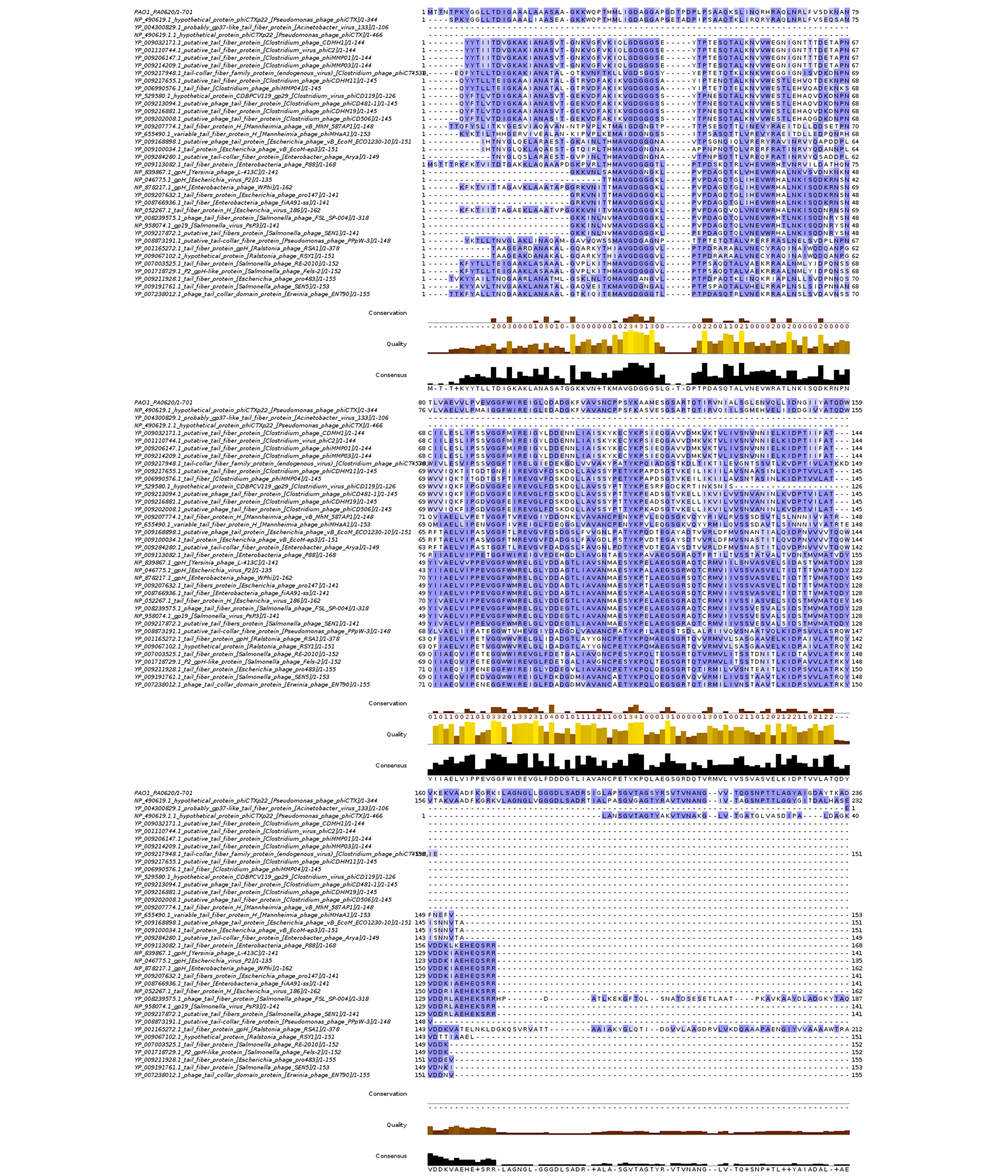

Supplement: S2 Fig — R2-type pyocin tail fiber sequence from P. aeruginosa, strain PAO1, was used as the subject for a pBLAST query against NCBI genomic database myophage genomes. Myophage genome hit ORFs were extracted, aligned by CLUSTAL Ω, and the resulting alignment rendered in JALVIEW. Individual sequences are colored from white (low conservation) to dark blue (high conservation). The alignment conservation score (0–9), relative quality of genomic data, and consensus sequence, appear as separate tracks with associated sequence positions. (TIF) [file pone.0211432.s002.tif]

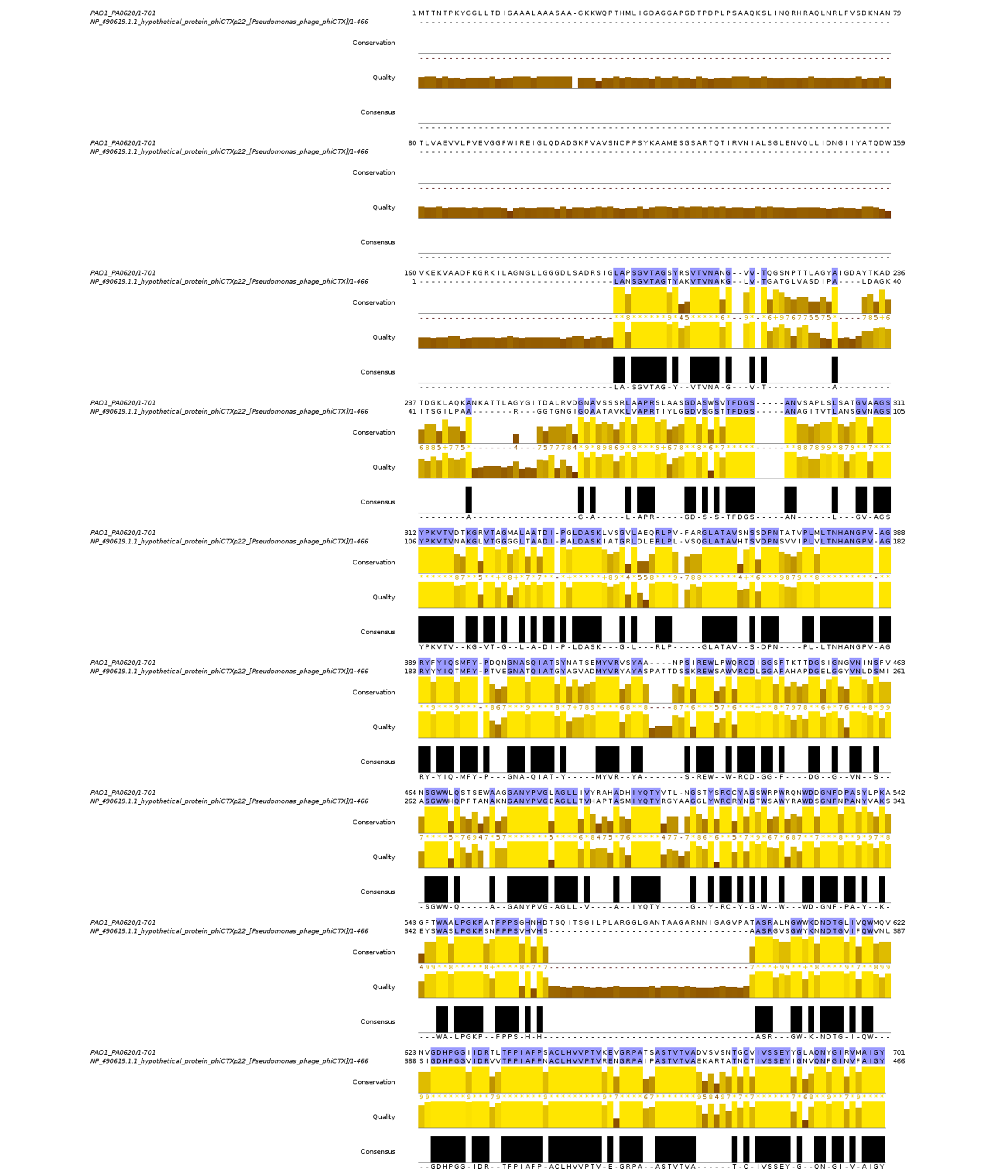

Supplement: S3 Fig — JALVIEW rendering of CLUSTAL Ω alignment between the R2-type pyocin tail fiber and ɸCTX contig containing the p22 tail fiber C-terminus. Alignment colored according to the BLOSUM62 convention. (TIF) [file pone.0211432.s003.tif]

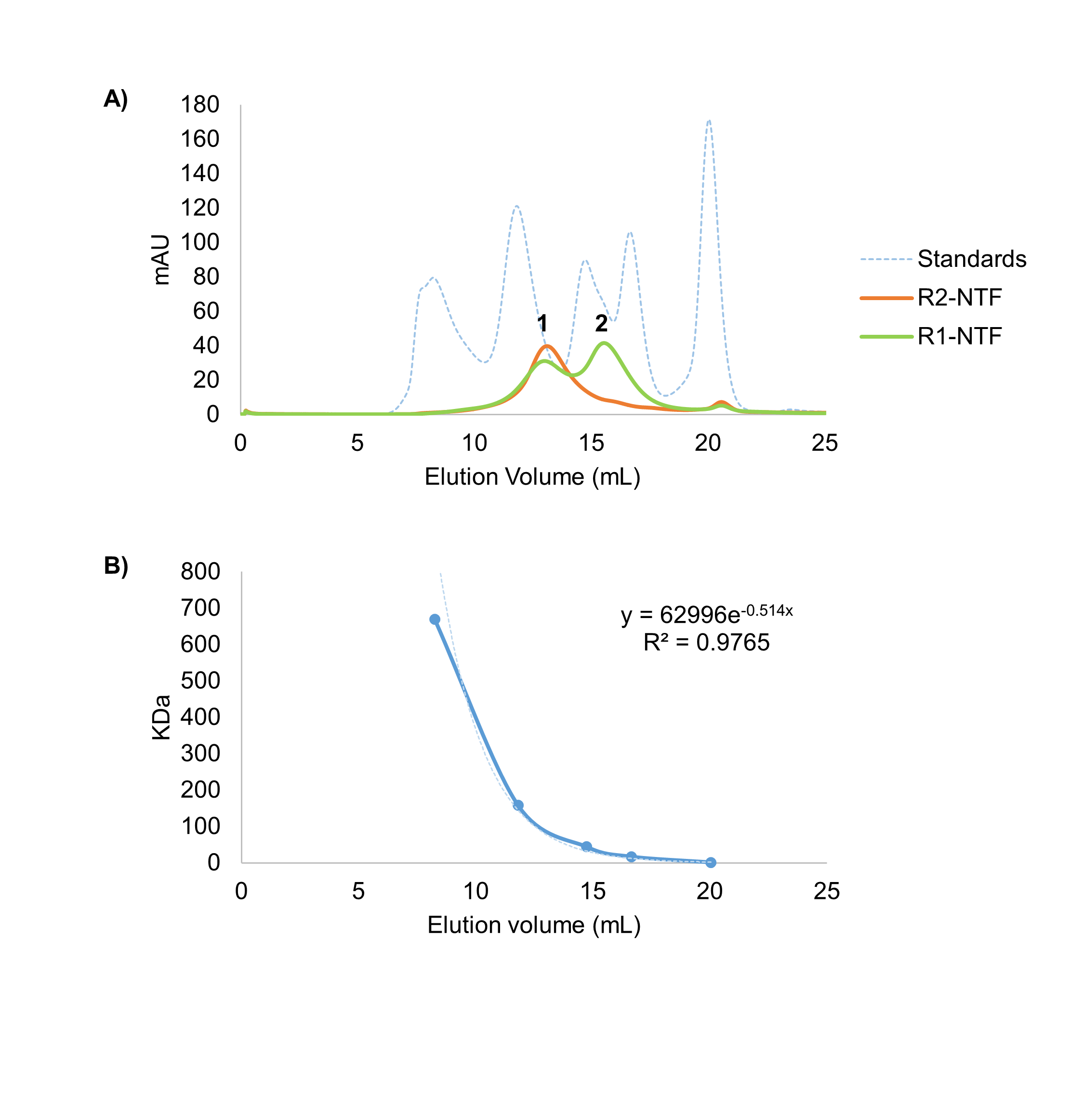

Supplement: S4 Fig — Analytical SEC reveals oligomeric state(s) of purified R1-NTF and R2-NTF. (A) UV chromatograph of R1-NTF (orange), R2-NTF (green), and SEC S-200 standards (blue dashes) showing peak elution times. (B) Plot of elution volume vs. molecular weight for SEC standards. Exponential regression reveals that peak 1 for the R2-NTF corresponds to a trimeric oligomer, and peak 2 for the R1-NTF corresponds to a dimer of trimers. (TIF) [file pone.0211432.s004.tif]

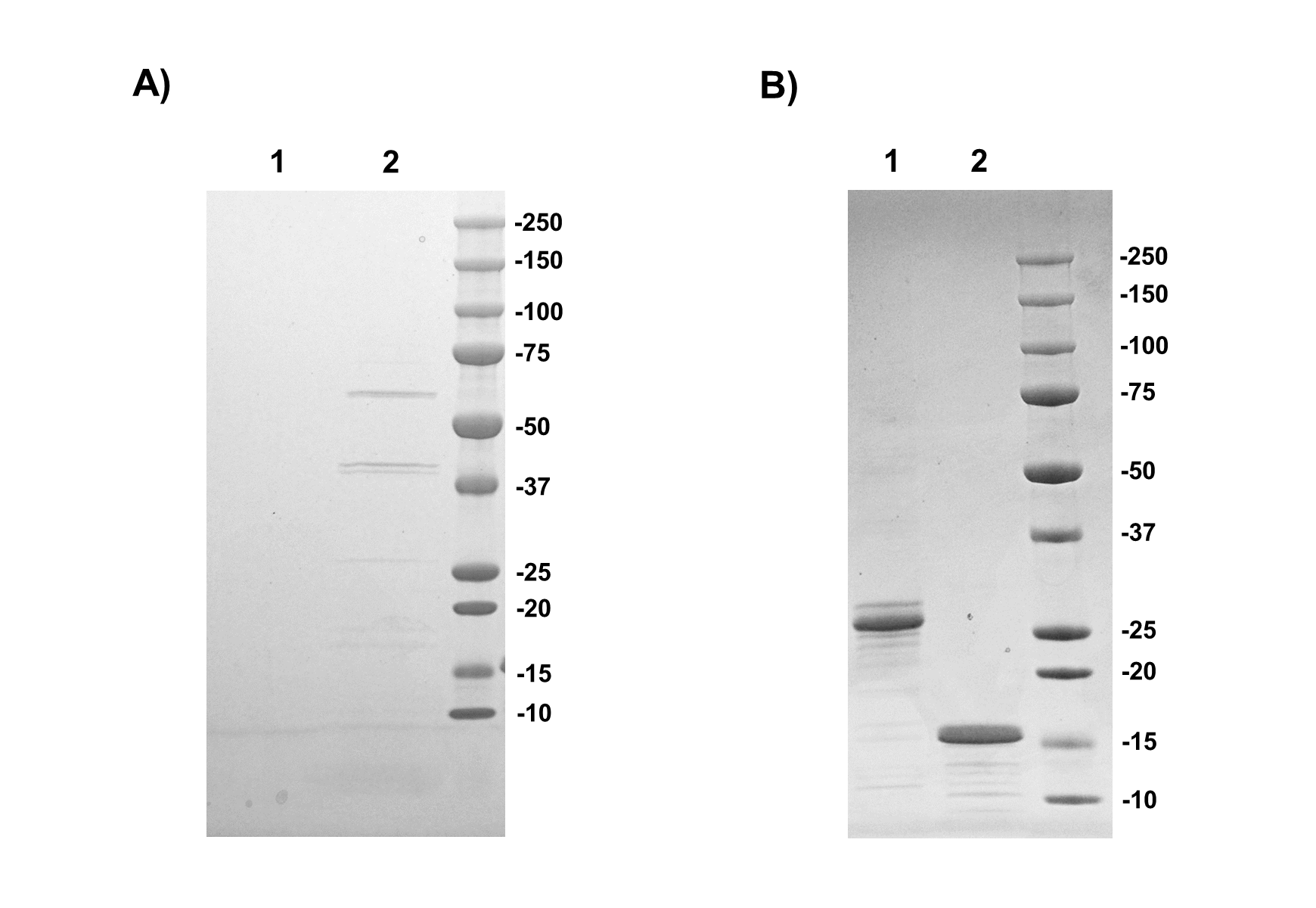

Supplement: S5 Fig — SDS-PAGE gels stained with Coomassie blue dye. (A) Purified R2-pyocin preparations (20μL); (lane 1) M9 buffer exchanged LB media. (lane 2) M9 buffer exchanged LB media with purified PAO1 LB supernatant. (lane 3) Precision plus protein standards, sizes listed in kDa. (B) Purified NTF samples used in crystallization and cell binding experiments; (lane 1), R2-NTF sample (1μL of 5mg/mL protein). (lane 2) R1-NTF sample (1μL of 5mg/mL protein). (lane 3) Precision plus protein standards, sizes listed in kDa. (TIF) [file pone.0211432.s005.tif]

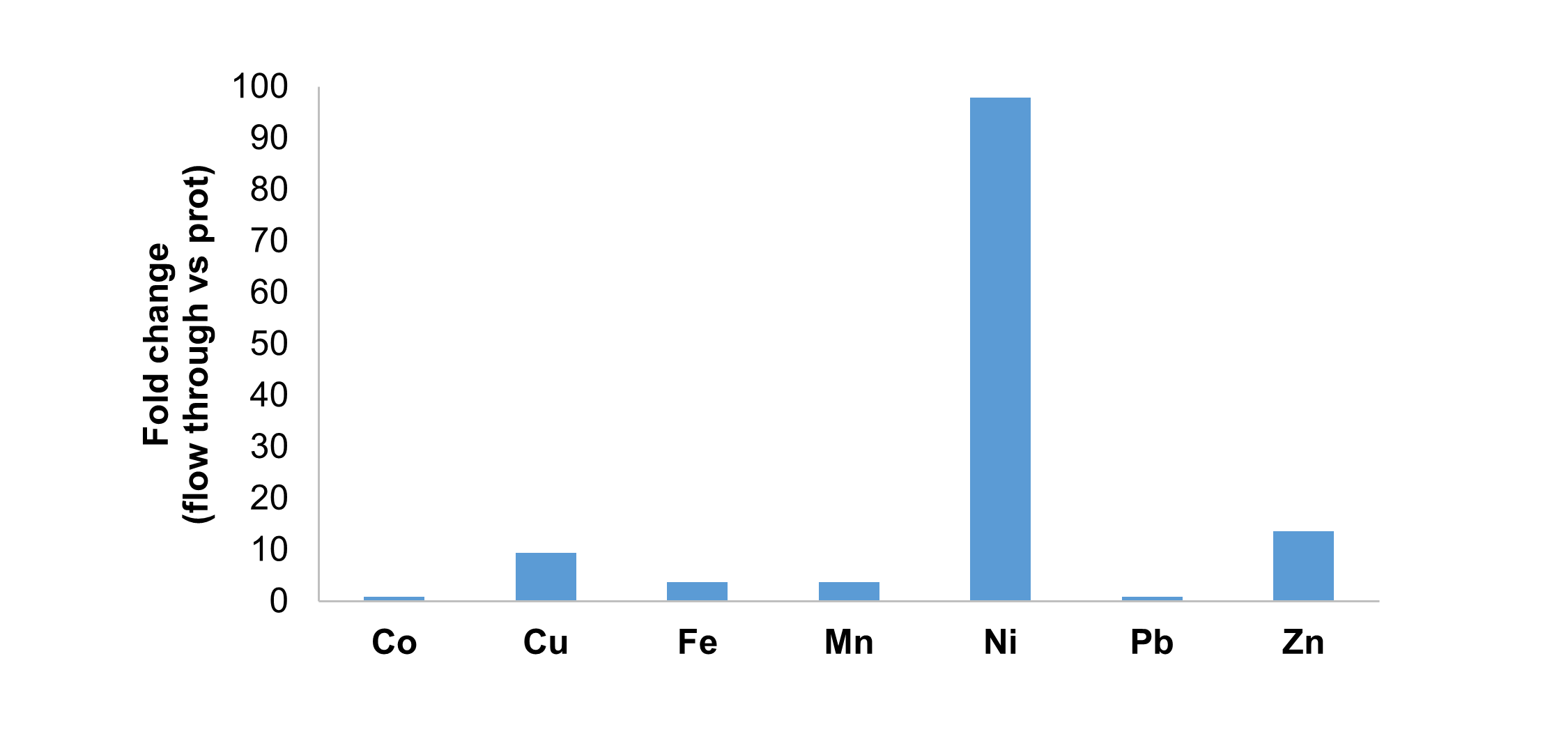

Supplement: S6 Fig — R2-NTF samples were buffer exchanged and concentrated to 1mg/mL. Fractions containing protein and concentrator flow-through were analyzed by ICP-MS for the presence of divalent cations. Plot of fold change between flow-through and protein fractions (n = 2). (TIF) [file pone.0211432.s006.tif]

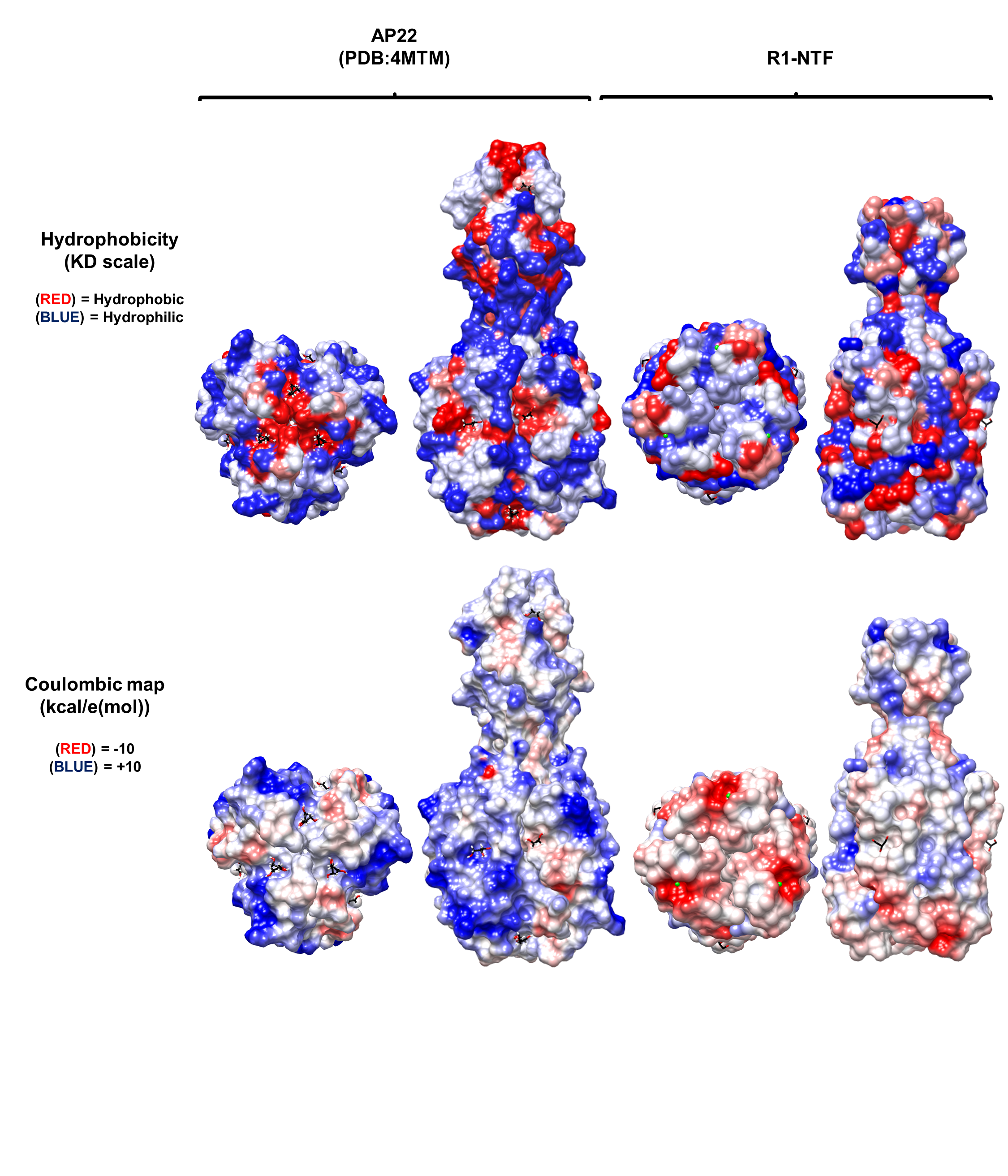

Supplement: S7 Fig — Hydrophobicity (top) or Charge scoring (bottom) of residues in the tail fiber of myophage AP22 (left) and R1-NTF (right). In AP22, ethanolamine and glycerol bind hydrophilic pockets absent in the R1-NTF structure. (TIF) [file pone.0211432.s007.tif]

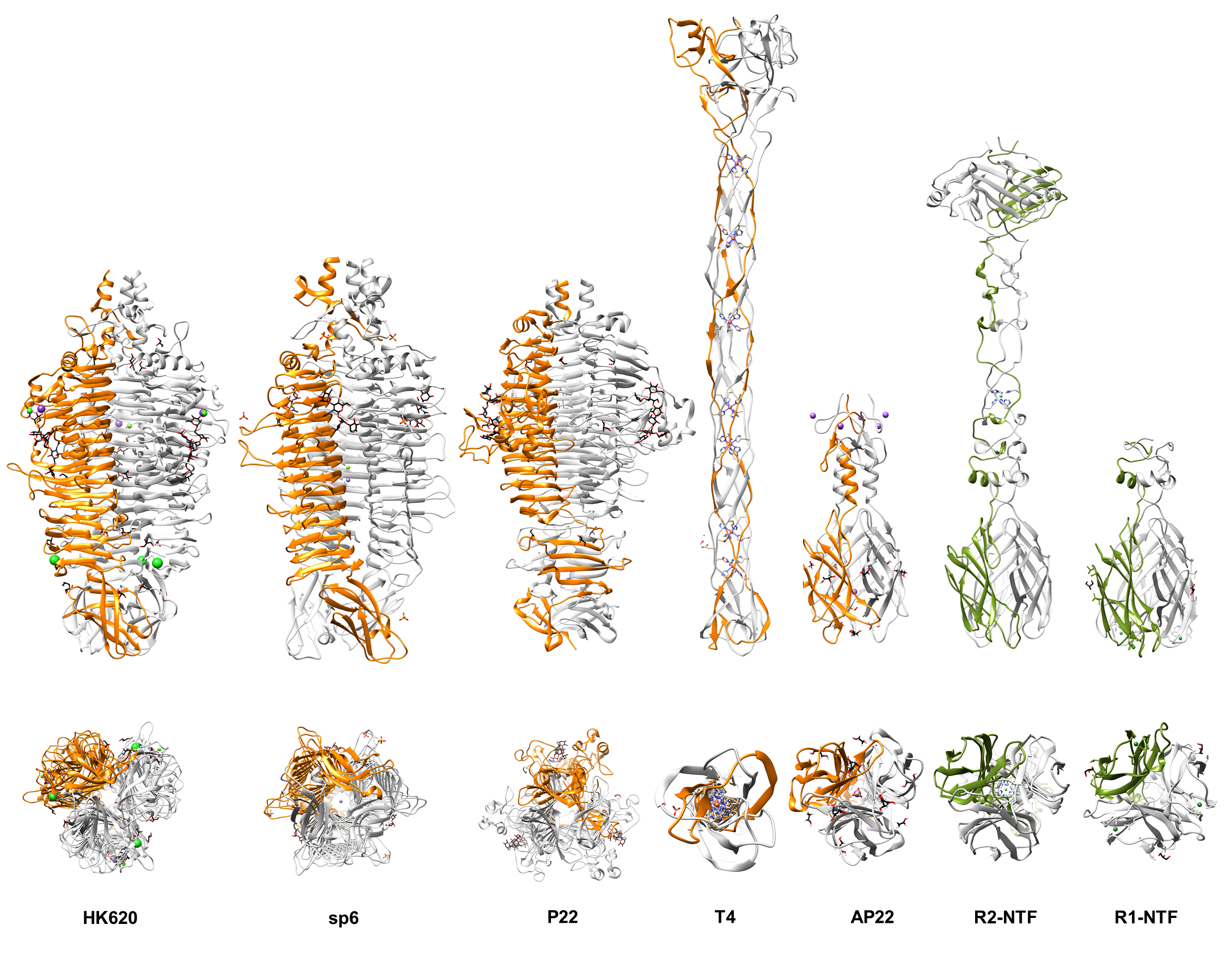

Supplement: S8 Fig — Structures of tail spikes and fibers compiled from the PDB and rendered in CHIMERA. (TIF) [file pone.0211432.s008.tif]
